# Anticoagulation, therapy of concomitant conditions, and early rhythm control therapy: a detailed analysis of treatment patterns in the EAST - AFNET 4 trial

**DOI:** 10.1093/europace/euab200

**Published:** 2021-09-02

**Authors:** Andreas Metzner, Anna Suling, Axel Brandes, , Günter Breithardt, A John Camm, Harry J G M Crijns, , Lars Eckardt, , Arif Elvan, , Andreas Goette, , Laurent M Haegeli, , Hein Heidbuchel, , Josef Kautzner, , Karl-Heinz Kuck, , Luis Mont, G Andre Ng, , Lukasz Szumowski, , Sakis Themistoclakis, , Isabelle C van Gelder, , Panos Vardas, , Karl Wegscheider, , Stephan Willems, Paulus Kirchhof

**Affiliations:** 1 Department of Cardiology, University Heart and Vascular Center, University Medical Center Hamburg-Eppendorf, Martinistraße 52, 20246 Hamburg, Germany; 2 German Center of Cardiovascular Research, Partner Site Hamburg/Lübeck/Kiel, Hamburg, Germany; 3 Institute of Medical Biometry and Epidemiology, University Medical Center Hamburg-Eppendorf, Hamburg, Germany; 4 Department of Cardiology, Odense University Hospital, Odense, Denmark; 5 Department of Clinical Research, University of Southern Denmark, Odense, Denmark; 6 Atrial Fibrillation Network (AFNET), Münster, Germany; 7 Department of Cardiology II (Electrophysiology), University Hospital Münster, Münster, Germany; 8 Cardiology Clinical Academic Group, Molecular and Clinical Sciences Research Institute, St. George’s University of London, London, UK; 9 Department of Cardiology, Maastricht University Medical Center and Cardiovascular Research Institute Maastricht, Maastricht, Netherlands; 10 Isala Hospital and Diagram Research, Zwolle, The Netherlands; 11 St. Vincenz Hospital, Paderborn, Germany; 12 Working Group of Molecular Electrophysiology, University Hospital Magdeburg, Magdeburg, Germany; 13 University Hospital Zurich, Zurich, Switzerland; 14 Division of Cardiology, Medical University Department, Kantonsspital Aarau, Aarau, Switzerland; 15 University Hospital Antwerp and Antwerp University, Antwerp, Belgium; 16 Institute for Clinical and Experimental Medicine, Prague, Czech Republic; 17 LANS Cardio, Hamburg, Germany; 18 University of Barcelona and Institut de Recerca Biomèdica, August Pi-Sunyer, Barcelona, Spain; 19 Centro Investigación Biomedica en Red Cardiovascular, Madrid, Spain; 20 Department of Cardiovascular Sciences, University of Leicester, National Institute for Health Research Leicester Biomedical Research Centre, Glenfield Hospital, Leicester, UK; 21 Arrhythmia Center of the National Institute of Cardiology, Medical Division of Cardinal Stefan Wyszynski University in Warsaw, Warsaw, Poland; 22 Department of Cardiology, Ospedale dell’Angelo, Venice, Italy; 23 University of Groningen, University Medical Center Groningen, Groningen, Netherlands; 24 Heart Sector, Hygeia Hospitals Group, Athens, Greece; 25 Department of Cardiology, Asklepios Klinik St. Georg (S.W.), Hamburg, Germany; 26 Cardiovascular Sciences, University of Birmingham, Birmingham, UK

**Keywords:** Atrial fibrillation, Anticoagulation, Rhythm control therapy, Antiarrhythmic drugs, Ablation, Stroke, Cardiovascular death, Heart failure

## Abstract

**Aims:**

Treatment patterns were compared between randomized groups in EAST-AFNET 4 to assess whether differences in anticoagulation, therapy of concomitant diseases, or intensity of care can explain the clinical benefit achieved with early rhythm control in EAST-AFNET 4.

**Methods and results:**

Cardiovascular treatment patterns and number of visits were compared between randomized groups in EAST-AFNET 4. Oral anticoagulation was used in >90% of patients during follow-up without differences between randomized groups. There were no differences in treatment of concomitant conditions between groups. The type of rhythm control varied by country and centre. Over time, antiarrhythmic drugs were given to 1171/1395 (84%) patients in early therapy, and to 202/1394 (14%) in usual care. Atrial fibrillation (AF) ablation was performed in 340/1395 (24%) patients randomized to early therapy, and in 168/1394 (12%) patients randomized to usual care. 97% of rhythm control therapies were within class I and class III recommendations of AF guidelines. Patients randomized to early therapy transmitted 297 166 telemetric electrocardiograms (ECGs) to a core lab. In total, 97 978 abnormal ECGs were sent to study sites. The resulting difference between study visits was low (0.06 visits/patient/year), with slightly more visits in early therapy (usual care 0.39 visits/patient/year; early rhythm control 0.45 visits/patient/year, *P* < 0.001), mainly due to visits for symptomatic AF recurrences or recurrent AF on telemetric ECGs.

**Conclusion:**

The clinical benefit of early, systematic rhythm control therapy was achieved using variable treatment patterns of antiarrhythmic drugs and AF ablation, applied within guideline recommendations.

**What’s new?**

- Early rhythm control therapy was delivered on top of high oral anticoagulation rates and high use of rate control in both randomized groups.
- There were no relevant differences in other cardiovascular treatments that could explain the outcome of the trial.
- Early rhythm control therapy was achieved with a very low number of study visits. Telemetric electrocardiogram (ECG) monitoring with over 300 000 transmitted ECG devices only resulted in approximately 150 extra visits in 1395 patients randomized to early rhythm control therapy (ca 0.09 visits/patient) over five years.
- Confirming other recent trials, the analysis demonstrates the safety of rhythm control therapy.
- The clinical benefit of early, systematic rhythm control therapy was achieved using variable treatment patterns of antiarrhythmic drugs and atrial fibrillation (AF) ablation, applied within guideline recommendations. These patterns can be followed to implement early rhythm control therapy for all patients with recently diagnosed AF and concomitant conditions.

## Introduction

Optimal management of patients with atrial fibrillation (AF) includes anticoagulation, rate control therapy, and therapy of concomitant cardiovascular conditions, which may be supplemented by rhythm control therapy in patients who remain symptomatic on optimal rate control according to current guidelines.^1^^,^^2^ Even on optimal therapy, patients with AF remain at high risk of cardiovascular death (1–2%/year),^3–6^ worsening of heart failure (3.5% of patients hospitalized for heart failure/year^4^^,^^5^^,^^7^), and stroke despite appropriate anticoagulation (1%/year^8^). Indeed, 5% of well-managed AF patients experience these severe complications per year.^6^^,^^9^

The EAST-AFNET 4 trial demonstrated that systematic, early initiation of rhythm control therapy results in a 21% relative risk reduction in a composite of cardiovascular death, stroke, and hospitalization for heart failure or acute coronary syndrome in a population of patients with recently diagnosed AF and concomitant cardiovascular conditions.^9^^,^^10^ The clinical benefit was achieved with equal overall safety, including fewer strokes, numerically lower mortality and more serious adverse events related to rhythm control therapy in patients randomized to early rhythm control. To provide context for this finding, and to enable delivery of early rhythm control therapy in clinical practice, the treatment patterns used in EAST-AFNET 4 need to be known in detail. Furthermore, unintended differences in the delivery of other components of AF therapy such as anticoagulation, therapy of concomitant cardiovascular conditions, or more intensive contacts with the study sites could have influenced the outcome of the study.

To increase understanding of the trial results and to enable their clinical implementation,^9^^,^^11^ treatment patterns were compared between randomized groups in the EAST-AFNET 4 trial population including anticoagulation, therapy of concomitant cardiovascular conditions, rate control therapy, study visits, and rhythm control therapy.

## Methods

This is a comparison of the treatment components between randomized groups in the EAST-AFNET 4 trial, and of the factors associated with specific therapies in the EAST-AFNET 4 dataset. The design of the EAST-AFNET 4 trial, the methods of analysis, and the main results have been published.^6^^,^^9^ The current analysis was performed on the final, locked database of the trial. Analyses included treatments at discharge from the randomization visit, at 1 year of follow-up, and at 2 years of follow-up. Descriptive data on the use of different therapies, including anticoagulation, therapy of concomitant cardiovascular conditions, rate control, and rhythm control therapy as well as the number of visits were summarized. In addition, therapies were classified as guideline-mandated based on the class I recommendations of ESC practice guidelines in use at the time.^2^^,^^12^^,^^13^ Treatment patterns were described and analysed for differences between randomized groups, clinical characteristics, and centre and country effects. Treatment changes over time were analysed and compared between randomized groups.

Continuous variables are reported as mean and standard deviation and categorical variables are presented as frequencies and percentages. For visualization, bar plots, box plots, and Aalen–Johansen cumulative incidence curves, accounting for the competing risk of death, were used. To determine the relation between administered rhythm control (antiarrhythmic drug, ablation, or none), anticoagulation therapy and potential factors (e.g. age, gender, country), we used mixed logistic regression models adjusted for the random effect of centre. Results are presented as odds ratios (ORs) together with 95% confidence intervals (CIs).

Mixed logistic regression models were also used to assess differences between treatment groups in the cardiovascular therapies, participation of main follow-up visits, and apparent violations of class I recommendations (or Fisher’s exact test if the mixed logistic regression model was not applicable). Mixed Poisson and mixed linear regression models were used to assess differences in the number of visits per patient and the number of visits per patient per year, respectively. Both model types were unadjusted and included a random term for the centre effect. All analyses were performed using STATA 16.1 (StataCorp. 2019) and R 4.0.2 (R Core Team 2020). The authors had access to the entire, locked database of the trial and vouch for the fidelity of the data and their analyses.

## Results

Between July 2011 and December 2016, 135 sites in 11 countries randomized 2789 patients to the EAST-AFNET 4 trial. Over half of the sites participating in EAST were smaller sites without on-site ablation facilities who cooperated with ablation centres. A total of 1752 patients (63%) were randomized in sites without on-site ablation facilities (called D-sites, Supplementary material online,*TableS1*), the remaining 1037 patients in sites performing AF ablation on-site (called A-sites). University hospitals randomized 579 (21%) patients, other hospitals 1276 (46%) patients, and office-based cardiologists 934 (33%) patients.

**Figure 1:**
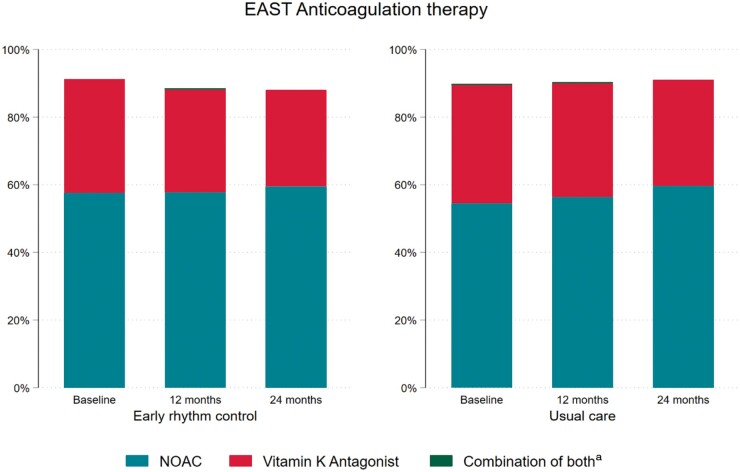
Anticoagulation therapy in patients randomized to early rhythm control (left panel) and usual care (right panel) in the EAST-AFNET 4 population at discharge from randomization, 1 year, and 2 years of follow-up. There was no difference in anticoagulation therapy between randomized groups. A combination of both was very rare and therefore the yellow bars are hardly visible. ^a^Combination of both can raise due to changes of medication between visits.

Over 90% of patients received guideline-mandated oral anticoagulation throughout the follow-up without differences between randomized groups (*Table 1*, Supplementary material online, *Table* *S2, Figure 1*). In a multivariate analysis, anticoagulation therapy at any time was influenced by patient’s age [OR 1.64, 95% CI (1.36–1.98); *P* < 0.001], gender [male vs. female OR 1.42, 95% CI (1.42, 95% CI (1.00–2.01); *P* = 0.048], and AF pattern [persistent or long-standing persistent vs. first episode or paroxysmal OR 3.38, 95% CI (1.81–6.31); *P* < 0.001], without differences between randomized groups (*P* = 0.912). The use of novel oral anticoagulants (NOACs) was high (>54% at baseline in both groups) with a slight further increase during follow-up.

**Table 1:** Cardiovascular therapies given to patients in the EAST-AFNET 4 trial at discharge from the baseline visit, at 12 months follow-up, and at 24 months follow-up

	Randomized group			
	Early rhythm control (*N* = 1395)	Usual care (*N* = 1394)	Total (*N* = 2789)	*P*-value
Patients receiving oral anticoagulation				
Anticoagulation (discharge from baseline)	1267/1389 (91.2%)	1250/1393 (89.7%)	2517/2782 (90.5%)	0.149
NOACs (discharge from BL)	800/1389 (57.6%)	763/1393 (54.8%)	1563/2782 (56.2%)	0.103
Vitamin K antagonists (discharge from BL)	467/1389 (33.6%)	490/1393 (35.2%)	957/2782 (34.4%)	0.397
Anticoagulation (12 months FU)	1087/1230 (88.4%)	1121/1241 (90.3%)	2208/2471 (89.4%)	0.111
NOACs (12 months FU)	713/1230 (58.0%)	704/1241 (56.7%)	1417/2471 (57.3%)	0.657
Vitamin K antagonists (12 months FU)	376/1230 (30.6%)	421/1241 (33.9%)	797/2471 (32.3%)	0.100
Anticoagulation (24 months FU)	1020/1159 (88.0%)	1065/1171 (90.9%)	2085/2330 (89.5%)	0.021
NOACs (24 months FU)	690/1159 (59.5%)	699/1171 (59.7%)	1389/2330 (59.6%)	0.774
Vitamin K antagonists (24 months FU)	330/1159 (28.5%)	366/1171 (31.3%)	696/2330 (29.9%)	0.202
Patients receiving rate control therapy (beta adrenoreceptor blocker, verapamil, diltiazem, or digitalis glycosides)				
Rate control (discharge from BL)	1088/1389 (78.3%)	1235/1393 (88.7%)	2323/2782 (83.5%)	<0.001
Rate control (12 months FU)	883/1230 (71.8%)	1055/1241 (85.0%)	1938/2471 (78.4%)	<0.001
Rate control (24 months FU)	799/1159 (68.9%)	986/1171 (84.2%)	1785/2330 (76.6%)	<0.001
Patients receiving any rate controlling medication (beta adrenoreceptor blocker, verapamil, diltiazem, digitalis glycosides, or antiarrhythmic drugs with rate controlling properties^a^)				
Patients receiving any rate controlling medication (discharge from BL)	1259/1389 (90.6%)	1250/1393 (89.7%)	2509/2782 (90.2%)	0.382
Patients receiving any rate controlling medication (12 months FU)	1065/1230 (86.6%)	1084/1241 (87.3%)	2149/2471 (87.0%)	0.588
Patients receiving any rate controlling medication (24 months FU)	968/1159 (83.5%)	1013/1171 (86.5%)	1981/2330 (85.0%)	0.042
Patients receiving diuretics				
Diuretics (discharge from BL)	559/1389 (40.2%)	561/1393 (40.3%)	1120/2782 (40.3%)	0.987
Diuretics (12 months FU)	508/1230 (41.3%)	521/1241 (42.0%)	1029/2471 (41.6%)	0.788
Diuretics (24 months FU)	478/1159 (41.2%)	507/1171 (43.3%)	985/2330 (42.3%)	0.299
Patients receiving heart failure and antihypertensive therapy (ACE inhibitor, angiotensin receptor blocker, mineralocorticoid antagonists, and neprilysin/valsartan)				
Heart failure and antihypertensive therapies (discharge from BL)	964/1389 (69.4%)	988/1393 (70.9%)	1952/2782 (70.2%)	0.397
Heart failure and antihypertensive therapies (12 months FU)	854/1230 (69.4%)	878/1241 (70.7%)	1732/2471 (70.1%)	0.482
Heart failure and antihypertensive therapies (24 months FU)	798/1159 (68.9%)	837/1171 (71.5%)	1635/2330 (70.2%)	0.163
Patients receiving diabetes therapy (oral antidiabetic medication and insulin)				
Antidiabetic therapy (discharge from BL)	256/1389 (18.4%)	254/1393 (18.2%)	510/2782 (18.3%)	0.873
Antidiabetic therapy (12 months FU)	238/1230 (19.3%)	237/1241 (19.1%)	475/2471 (19.2%)	0.870
Antidiabetic therapy (24 months FU)	228/1159 (19.7%)	227/1171 (19.4%)	455/2330 (19.5%)	0.924
Patients receiving statins				
Statins (discharge from BL)	628/1389 (45.2%)	568/1393 (40.8%)	1196/2782 (43.0%)	0.016
Statins (12 months FU)	587/1230 (47.7%)	526/1241 (42.4%)	1113/2471 (45.0%)	0.006
Statins (24 months FU)	576/1159 (49.7%)	529/1171 (45.2%)	1105/2330 (47.4%)	0.020

All patient numbers are given split by randomized group and in total. Proportions indicate proportions of patients receiving each therapy at each time point as a fraction of the totality of patients still in follow-up and with available medication information at that time point. Anticoagulation, therapy with heart failure and antihypertensive drugs, antidiabetic therapy, and rate control therapy were used in most patients.

ACE inhibitor, angiotensin-converting enzyme inhibitor; BL, baseline visit; FU, follow-up; NOAC, novel oral anticoagulant.

a Antiarrhythmic drugs with rate controlling properties are amiodarone, dronedarone, propafenone, and sotalol. *P*-values resulting from mixed logistic regression with centre as random effect.

**Figure 2:**
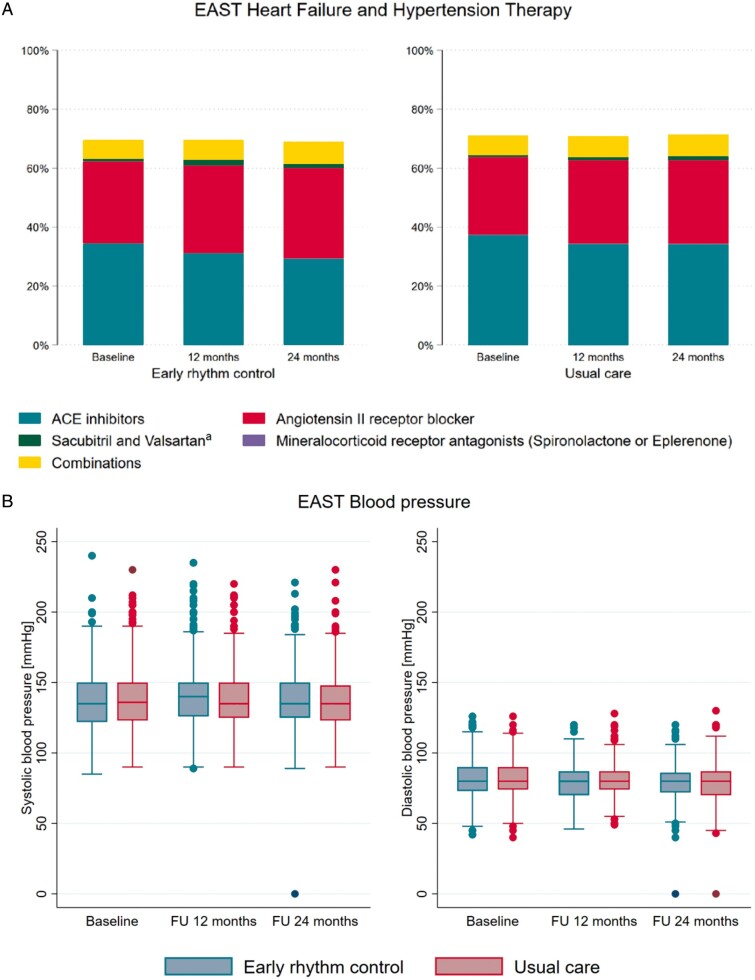
(*A*) Use of inhibitors of the renin–angiotensin–aldosterone system in patients randomized to early rhythm control (*left panel*) and usual care (*right panel*) in the EAST-AFNET 4 population.(*B*) Systolic and diastolic blood pressure during the in-person visits, split by randomized groups. Blood pressure was not different between randomized groups. ^a^All Sacubitril and Valsartan are given only in combination with other medications.

Therapy of concomitant cardiovascular conditions appeared well balanced, with about 70% of patients receiving inhibitors of the renin-angiotensin-aldosterone system. Blood pressure was not different between randomized groups throughout follow-up (*Table 1*, Supplementary material online, *Table* *S2, Figure 2*).

**Figure 3:**
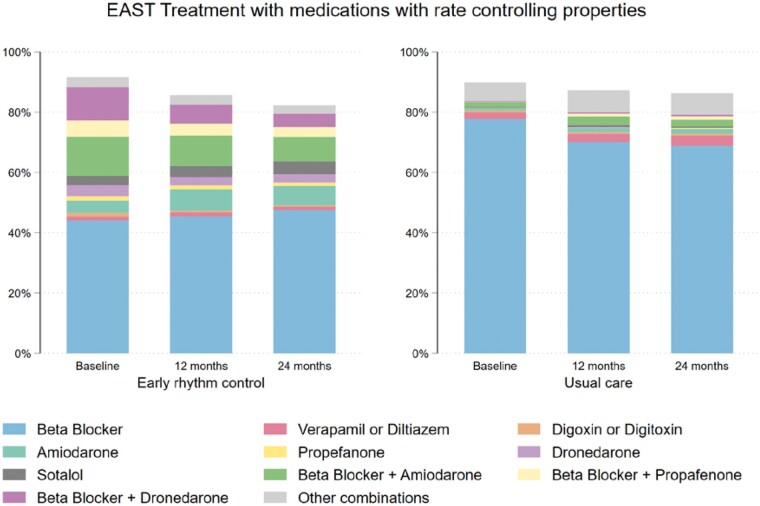
Use of any rate controlling therapies in patients randomized to early rhythm control (*left panel*) and usual care (*right panel*) in the EAST-AFNET 4 population. This display includes antiarrhythmic drugs with rate controlling properties, namely amiodarone, dronedarone, propafenone, and sotalol. The use of these medications often obviates the need for additional rate-controlling medication, explaining the lower use of beta blockers, calcium channel antagonists, or digoxin shown in *A*.

Rate control therapy was used in most patients. Overall, 1088/1389 (78.3%) patients randomized to early rhythm control therapy received beta-blockers, verapamil or diltiazem, or digitalis glycosides at discharge from the baseline visit, and 1235/1393 (88.7%) patients randomized to usual care. When the use of antiarrhythmic drugs with rate controlling properties (amiodarone, dronedarone, propafenone, or sotalol) was included in the analysis, the difference in rate control was much less pronounced (*Table 1*, *Figure 3*). The use of rate control decreased during follow-up in both groups, more in patients randomized to early rhythm control.

**Figure 4:**
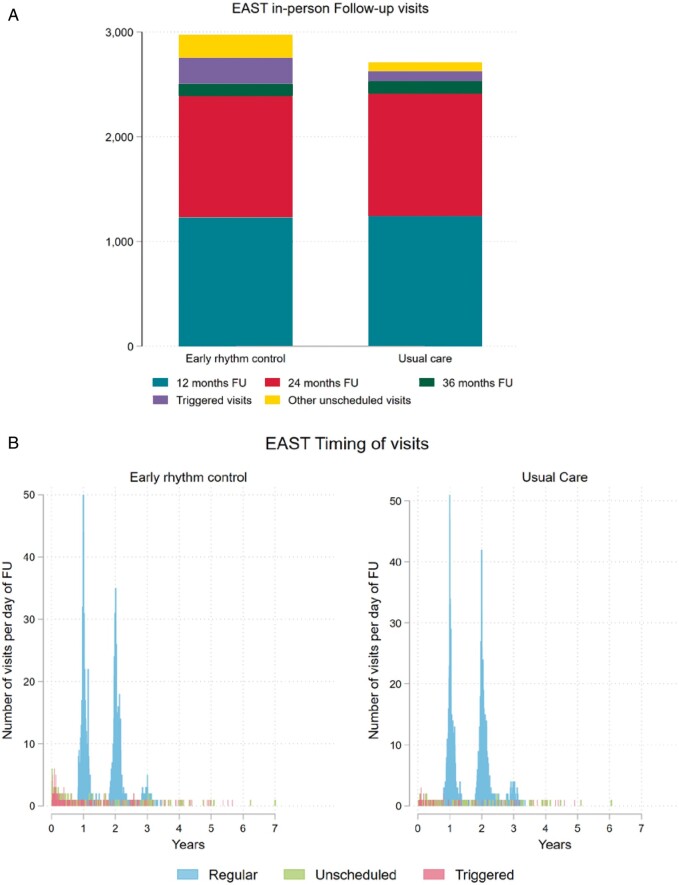
(*A*) Number of in-person visits split by randomized group. There were 2710 in-person visits in patients randomized to usual care (1.94 visits/patient) and 2974 in-person visits in patients randomized to early rhythm control (2.13 visits/patient) (*P* < 0.001). (*B*) Timing of in-person follow-up visits split by randomized group and by visit type. All numbers are displayed as number of visits per day.

In-person visits were infrequent during the median follow-up of 5.1 years per patient due to the study design.^6^ Patients randomized to early rhythm control therapy underwent 2974 in-person visits (2.13/patient, 0.45 visits/patient/year) including 249 visits triggered by detection of recurrent AF calling for an adjustment of rhythm control therapy (so-called triggered visits),^6^ slightly more than the 2710 visits (1.94/patient, 0.39 visits/patient/year) including 93 triggered visits in patients randomized to usual care (*Table 2*, *Figure 4A*). The increase in site visits seen in patients randomized to early therapy was mainly driven by triggered visits to adjust rhythm control therapy (*Figure 4B*). Patients randomized to early therapy transmitted 297 166 telemetric, 30-s electrocardiogram (ECG) recordings to a core lab. Of these, 97 978 were judged as abnormal and sent to study sites for review and to decide on clinical consequences. Only a small number of abnormal telemetric ECGs led to clinical actions: Of the 249 triggered visits performed in patients randomized to early rhythm control, approximately 150 were due to abnormal telemetric ECGs.

**Table 2:** In-person study visits at 1, 2, and 3 years, triggered and unscheduled visits

	Early treatment	Usual care	*P*-value
FU 12 months	1230	1241	0.495^a^
FU 24 months	1159	1171	0.545^a^
FU 36 months	117	119	0.849^a^
Triggered visits total (nr. per patient)	249 (0.18)	93 (0.07)	<0.001^b^
Unscheduled visits total (nr. per patient)	219 (0.16)	86 (0.06)	<0.001^b^
Total number of visits total (nr. per patient)	2974 (2.13)	2710 (1.94)	<0.001^b^

FU, follow-up.

a *P*-value resulting from mixed logistic regression.

b *P*-value resulting from mixed Poisson regression; both models with centre as random effect.

**Figure 5:**
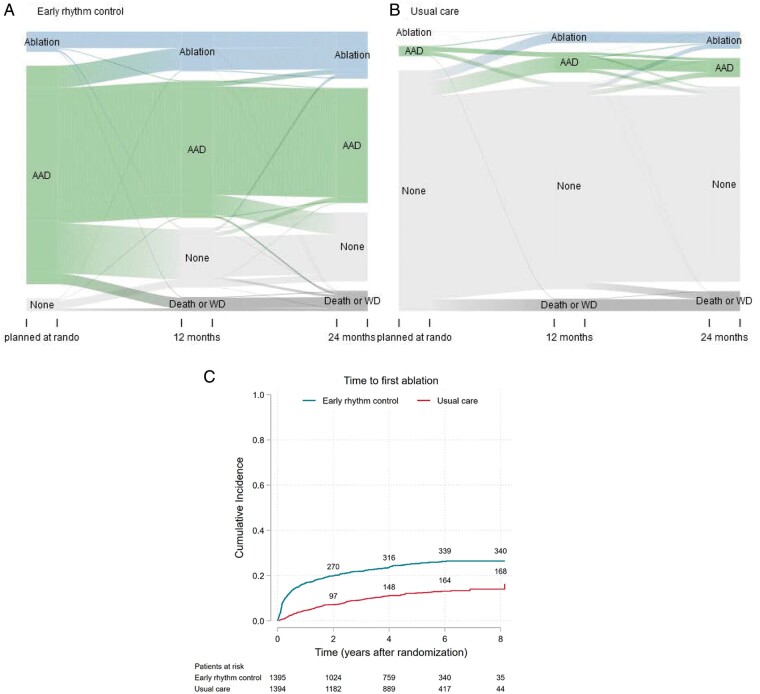
(*A*) Sankey Plot of rhythm control treatment over time per group. Shown is the proportion of patients receiving antiarrhythmic drugs (AAD) and AF ablation (ablation) at each of the scheduled visits, split by randomized groups, and the proportion of patients changing from one type of therapy to the other. (*B*) Time to first AF ablation split by randomized group (Aalen–Johansen cumulative incidence curve). AF ablation was more often used in patients randomized to early therapy, with a steady increase in both randomized groups over time. At 2 years, 270/1395 (19.4%) patients randomized to early therapy had undergone AF ablation, while 97/1394 (7.0%) patients randomized to usual care had undergone ablation.

**Figure 6:**
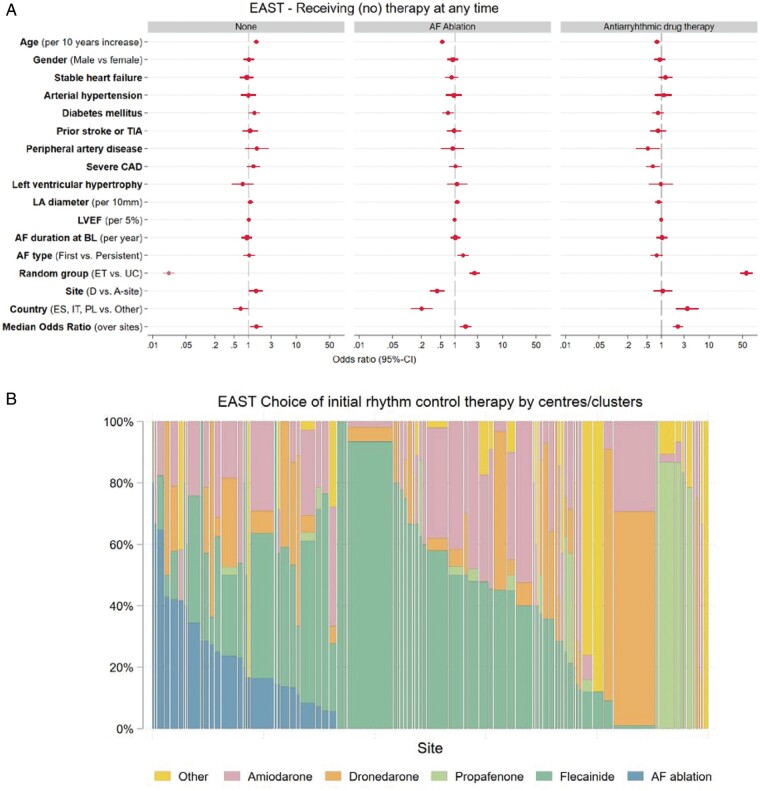
(*A*) Multivariate analysis of potential factors influencing the decision to manage patients without rhythm control therapy (None, *left panel*), to perform AF ablation (*middle panel*), and to initiate antiarrhythmic drug therapy (AAD, *right panel*) at any time. The decision to manage without rhythm control therapy was almost entirely driven by randomized group. The decision to perform AF ablation was also influenced by younger age, randomization in an ablation site, diabetes, AF pattern, and country. AF type first, first episode or paroxysmal, persistent, persistent or long-standing persistent; ET, early treatment; Left ventricular hypertrophy on echocardiography was defined based on the inclusion criterium (>15 mm wall thickness); Severe CAD, severe coronary artery disease (previous myocardial infarction, CABG, or PCI); Stable heart failure was defined as either NYHA stage II or LVEF < 50%; TIA, transient ischaemic attack; UC, usual care. (*B*) Choice of initial rhythm control therapy displayed by centre. Displayed is the proportion of patients receiving each rhythm control therapy option in each centre, limited to centres that initiated rhythm control therapy in at least five patients. There are clear centre-based preferences in the choice of initial antiarrhythmic drug therapy, with individual sites using AF ablation, flecainide, propafenone, dronedarone, or other antiarrhythmic drugs in most patients initially. Therapy choices were guideline-conform in almost all patients.

Of 2789 patients, 508 patients (18.2%) received an ablation at any time, with 340/1395 (24%) patients randomized to early therapy receiving ablation. Antiarrhythmic drug therapy was given to 1373 (49.2%) patients, including 1171/1395 (84%) of those randomized to early therapy. A total of 1208/2789 (43.3%) were managed without ablation or antiarrhythmic drug therapy throughout the trial [usual care: 1079/1394 (77%)]. Almost all patients (>97% of those receiving rhythm control therapy) received rhythm control therapy aligned with the class I recommendations in guidelines (*Table 3*). Some centres preferentially used AF ablation for rhythm control management, reflecting access to therapy and preferences by the local study teams. Others preferentially used flecainide, propafenone, dronedarone, or other amiodarone as initial rhythm control therapy in the majority of their patients. Adjustments to rhythm control therapy were relatively common in the first year after randomization, predominantly in patients randomized to early rhythm control (*Figure 5A*). Many ablations were performed immediately following randomization to early rhythm control. Thereafter, the number of patients treated by ablation increased steadily in both randomized groups (*Figure 5B*). At 2 years, 270/1395 (19.4%) patients randomized to early rhythm control therapy had undergone AF ablation, while 97/1394 (6.9%) patients randomized to usual care had undergone ablation. This corresponded to 26.7% of patients still in follow-up at 2 years. The decision to manage a patient without rhythm control therapy was almost exclusively explained by randomized group without any relevant other effects [OR early treatment vs. usual care 0.02, 95% CI (0.02; 0.03); *P* < 0.001], *Figure 6A*, Supplementary material online, *Table* *S4*. The initial choice of the type of rhythm control therapy varied by centre (*Figure 6B*). AF ablation was more likely given to patients randomized to early treatment, patients recruited in an A-site or in another country than Spain, Italy, or Poland, younger patients, those without diabetes mellitus, and patients included with first diagnosed or paroxysmal AF (*Figure 6* and Supplementary material online, *Table* *S3*). If patients randomized to usual care remained symptomatic despite optimal rate control, the trial protocol called for rhythm control initiation by means of antiarrhythmic drugs or ablation. The high proportion of patients without AF-related symptoms (EHRA I) in both randomized groups at two years substantiates the adequate, protocol-conform use of rhythm control to improve AF-related symptoms in the usual care arm.

**Table 3:** Apparent violations of class I recommendations for rhythm control therapy use in the EAST-AFNET 4 population

	Randomized group			
	Early rhythm control (*N* = 1395)	Usual care (*N* = 1394)	Total (*N* = 2789)	*P*-value
Severe coronary artery disease in patients receiving flecainide or propafenone at discharge	32 (2.3%)	3 (0.2%)	35 (1.3%)	<0.001^a^
Reduced left ventricular function in patients receiving flecainide or propafenone at discharge	2 (0.1%)	1 (0.1%)	3 (0.1%)	0.572^a^
Reduced left ventricular function in patients receiving dronedarone at discharge	3 (0.2%)	0 (0.0%)	3 (0.1%)	0.250^b^
At least one violation of guideline conform use	37 (2.7%)	4 (0.3%)	41 (1.5%)	<0.001^a^

Over 97% of patients received rhythm control therapy in line with recommendations of the ESC guidelines published between 2012 and 2020.^1^^,^^10^^,^^11^ The most common apparent violation was the use of sodium channel blockers in patients with coronary artery disease (35 patients, 1.3%).

a *P*-value resulting from mixed logistic regression with centre as random effect.

b *P*-value resulting from Fisher’s exact test.

## Discussion

### Main findings

This in-depth analysis of the therapies given to patients participating in the EAST-AFNET 4 trial produced three major results.

1. A strategy of systematic and early rhythm control therapy achieved clinical benefit when added to evidence-based anticoagulation and rate control therapy.
2. There were no relevant differences in other cardiovascular treatments that could explain the outcome of the trial.
3. EAST-AFNET 4 implemented early rhythm control without many additional visits: On average, each patient was seen 1.94 (usual care) and 2.13 (early therapy) times by the study centre during the follow-up of approximately 5 years.
4. Early, systematic rhythm control was achieved using a combination of antiarrhythmic drugs and AF ablation. Early rhythm control treatment patterns varied by site and country within guideline recommendations, outlining a range of ways to provide early rhythm control therapy to patients with AF.

### Anticoagulation

Evidence-based anticoagulation use was high (>90% throughout follow-up) without differences between randomized groups. Approximately half of the patients were treated with NOACs at discharge from randomization, increasing slightly at 2 years (*Table 1*), comparable to concomitant and more recent large European observational data sets.^14^^,^^15^ The adequate, continued use of anticoagulants and the high therapy adherence can explain the low stroke rate observed in EAST-AFNET 4,^9^ consistent with reports from large anticoagulation trials, and different from the AFFIRM trial.^4^^,^^16^

*Concomitant cardiovascular conditions* were treated without differences between randomized groups. Blood pressure, an important surrogate outcome associated with stroke and other cardiovascular events, was not different between randomized groups. There were 4–6% more patients randomized to early therapy who received statins. While this difference was significant, and can contribute to a reduction in acute coronary syndrome, stroke, and even cardiovascular death, it is very small. In view of the balanced distribution of therapies for other cardiovascular comorbidities, the lack of differences in blood pressure between randomized groups, and in view of the long-term outcomes of RACE-3,^17^ where a randomized intervention with high use of statins, Mineralocorticoid receptor antagonists (MRAs), and nurse-led care did not improve five-year outcomes (neither for recurrent AF nor for MACCE, recently presented at EHRA 2021), it is unlikely that undetected differences in this treatment domain can explain the differences in outcomes observed in EAST-AFNET 4.

*Rate control therapy* was given to the vast majority of patients in EAST-AFNET 4, in line with current guidelines (*Table 1*). Digoxin was used in a very small number of patients, and almost entirely as second-line therapy on top of beta-blockers, following current recommendations and trial results.^18^ Whether this remains best practice in patients with AF and heart failure remains to be tested in light of the recently published RATE-AF trial.^19^

### Number of visits

The number of study visits was low in both study arms, but slightly and significantly higher in patients randomized to early therapy (usual care 0.39 visits/patient/year, early rhythm control 0.45 visits/patient/year, *P* < 0.001). As can be appreciated in *Figure 4*, most of these visits occurred early after randomization, most likely to adjust rhythm control therapy. The number of extra visits induced by telemetric ECG monitoring is lower than expected at the start of the trial.^6^ As the results of abnormal telemetric ECG recordings were only revealed to study sites,^6^ this small increase in study visits will capture almost all additional visits induced by telemetric ECG monitoring (*Table 2*, *Figure 4A*). Together with the reported finding that there was no difference in nights spent in hospital between groups,^8^ these data demonstrate that early therapy was delivered with few added visits, and that differences in the intensity of care between groups cannot explain the observed effects of early therapy on cardiovascular death, stroke, and hospitalizations for heart failure or acute coronary syndrome. While delivery of care in a controlled trial will differ from routine clinical care, the excellent delivery of all domains of AF care in the EAST-AFNET 4 centre networks with few planned or unplanned visits may provide exemplars for the delivery of holistic, integrated, cost-effective care for patients with AF.

*Rhythm control therapy* was well aligned with guidelines, with >97% of control therapies following accepted class I recommendations (*Table 3*).^2^^,^^12^ Early rhythm control was initially delivered as antiarrhythmic drug therapy in most patients, and ¾ of patients were treated without AF ablation throughout the trial. AF ablation was used in ca ¼ of patients randomized to early therapy (*Figure 5B*), illustrating the importance of this treatment modality in the trial. As expected for early rhythm control, the difference between the use of AF ablation was most marked in the first few months after randomization (*Figure 5B*). In patients randomized to usual care, rhythm control was used in 15% of patients at 2 years, very similar to general AF registries reporting rhythm control^15^^,^^20^^,^^21^ and at a rate anticipated in the design of the trial.^5^ In addition to randomization to early rhythm control, the use of AF ablation was associated with enrolment at an A-site, younger age, no diabetes mellitus, and with first diagnosed or paroxysmal AF (*Figure 6A*). Furthermore, there were regional differences in the use of AF ablation, probably reflecting the access to AF ablation at the time of enrolment into the trial (2011–2016). Furthermore, regional differences in the competence and practice of antiarrhythmic drug therapy probably drove these differences.

Sinus rhythm rates were higher on early rhythm control in EAST-AFNET 4 (80% at 2 years^8^) than in AFFIRM^22^ or AF-CHF,^23^ illustrating the effectiveness of the early therapy strategy. The high rate of sinus rhythm in the early treatment arm might be explained by the modern rhythm control therapy patterns including safe use of sodium channel blockers, treatment with dronedarone, and AF ablation. These components of rhythm control therapy were not available at the time of AFFIRM and only rarely use in AF-CHF. The early timing of rhythm control therapy can furthermore explain the high rate of sinus rhythm.^24^

### Treatment patterns used to deliver early rhythm control therapy

EAST-AFNET 4 was a strategy trial. The vast majority of the rhythm control therapy options used in EAST-AFNET 4 (ca 97%, *Table 3*) are supported by AF treatment guidelines^2^^,^^12^ and led to few safety events due to antiarrhythmic drug or AF ablation.^3^^,^^25^ EAST-AFNET 4 enrolled patients from 2011 to 2016. While the use of AF ablation was high for the practice at the time, it seems likely that contemporary rhythm control therapy may make more use of AF ablation in light of recent data illustrating its safety,^3^^,^^25^ improvement in quality of life,^26^^,^^27^ and effectiveness in maintaining sinus rhythm.^28^^,^^29^

A high degree of centre-based variation was found in the initial selection of rhythm control therapy. This is in keeping with reports from the Veterans Administrations database where centre-based effects were a key determinant of the choice of antiarrhythmic drug.^25^ Possible drivers of these differences are local experience, protocols, access to therapy options, reimbursement, and others.^30^ The clinical benefit of early rhythm control was not affected by type of centre, underpinning that different treatment patterns can be used to achieve early rhythm control. Important for the interpretation of the trial is that all centres had access to AF ablation performed in experienced centres.

The current analysis emphasizes the relevance of AF ablation for safe and effective rhythm control therapy, used in a quarter of patients randomized to early rhythm control therapy, but also the effectiveness of antiarrhythmic drugs when initiated early, sufficient in around 75% of patients to deliver early rhythm control therapy. It is likely that sinus rhythm, lack of documented or symptomatic AF recurrences, failure of rhythm control, and patient preferences were the drivers of discontinuation of rhythm control therapy during the course of the study in circa 35% of patients randomized to early rhythm control at 2 years (*Figure 5A*).

### Limitations

While the EAST-AFNET 4 trial enrolled almost 3000 patients in 11 European countries with different healthcare systems, actively enrolling in sites with and without on-site AF ablation, small cardiology practices and large tertiary care centres, reflecting different treatment patterns and cultures, there may be further, different rhythm control treatment patterns with equal effectiveness. It is likely that different patterns and potentially different outcomes could arise from contemporary delivery of rhythm control, e.g. more AF ablations. It is unclear whether differences in therapy choices had an effect on outcomes. This requires complex modelling that is beyond the scope of this analysis.

## Conclusions

Different patterns of early rhythm control therapy resulted in lower rates of cardiovascular death, stroke, and hospitalizations for heart failure or acute coronary syndrome when added to a comprehensive management of AF including anticoagulation, therapy of concomitant cardiovascular conditions, and rate control therapy. There were no differences between randomized groups other than the study intervention that could explain the difference in clinical outcomes. Early rhythm control was delivered using different treatment patterns, providing a range of choices how to deliver early rhythm control therapy to achieve clinical benefit in patients with AF.

## Supplementary material

Supplementary material is available at *Europace* online.

## Funding

This work was funded by the AFNET, DZHK, EHRA, DHS, Abbott Laboratories, Sanofi; EAST-AFNET 4 ISRCTN number, ISRCTN04708680; ClinicalTrials.gov number, NCT01288352; EudraCT number, 2010-021258-20. PK is partially supported by European Union BigData@Heart (grant agreement EU IMI 116074), British Heart Foundation (FS/13/43/30324; PG/17/30/32961 and PG/20/22/35093; AA/18/2/34218), German Centre for Cardiovascular Research supported by the German Ministry of Education and Research (DZHK), and Leducq Foundation.

**Conflict of interest:** G.B. and P.K.: grants or support for AFNET from DZHK, EHRA, DHS, Abott Laboratories, Sanofi. A. S. and K.W.: grant from AFNET for statistical analysis. AJC received an institutional grant from Abbott and personal fees from Abbott, Boston Scientific, Medtronic and Sanofi. AB reports personal fees from Bayer, personal fees from Boehringer Ingelheim, grants from Biotronik, personal fees from Bristol-Myers Squibb, grants from Theravance, outside the submitted work. AG receives all support for the present manuscript from AFNET, Sanofi-Aventis, St. Jude Medical; grants or contracts from EU Horizon 2020; Grant No. 965286; consulting fees from Berlin Chemie, Boston Scientific, Medtronic, Omeicos, Daiichi Sankyo and payment or honoraria for lectures, presentations, speakers bureaus, manuscript writing or educational events: Bayer, BMS, Pfizer, Boehringer Ingelheim, Berlin Chemie, Boston Scientific, Medtronic, Menarini. AM reports personal fees from Medtronic, personal fees from EPD, personal fees from Biosense Webster, outside the submitted work. AS reports grants from AFNET during the conduct of the study; grants from BIOTRONIK, outside the submitted work. GAN reports grants from Boston Scientific, grants and personal fees from Abbott, personal fees from Biosense Webster, personal fees from Catheter Precision, personal fees from Daiichi Sankyo, outside the submitted work. GB reports grants to AFNET for the EAST Trial from Sanofi-Aventis, grants from Abbott Vascular, grants from BMBF (German Ministry of Education and Research, Grant Number: 01 GI 0204), grants from DZHK (German Centre for Cardiovascular Research), grants from EHRA (European Heart Rhythm Association, a branch of the European Society of Cardiology), grants from Deutsche Herzstiftung (German Heart Foundation), during the conduct of the study. Outside the submitted work: personal fees from Boehringer Ingelheim, BMS, Bayer Health Care, Johnson & Johnson, Sanofi-Aventis, Portola, Biosense, Biotronik, Daiichi Sankyo, and grants to AFNET from BMS and Biosense. HH reports grants from Abbott, grants from Medtronic, grants and personal fees from Biotronik, grants from Boston-Scientific, grants from Bayer, grants from Boehringer-Ingelheim, grants from Daiichi-Sankyo, grants and personal fees from Pfizer-BMS, outside the submitted work. HJGMC reports Netherlands Cardiovascular Research Initiative: an initiative with support of the Dutch Heart Foundation, CVON 2014-9: Reappraisal of Atrial Fibrillation: interaction between hyperCoagulability, Electrical remodeling, and Vascular destabilisation in the progression of AF (RACE V). IVG reports financial support by the Netherlands Cardiovascular Research Initiative: an initiative with support of the Dutch Heart Foundation, CVON 2014-9: Reappraisal of Atrial Fibrillation: interaction between hyperCoagulability, Electrical remodeling, and Vascular destabilisation in the progression of AF (RACE V). JK reports personal fees from Boehringer Ingelheim, grants and personal fees from Biosense Webster , personal fees from Biotronik, personal fees from Boston Scientific, grants from Affera inc, grants and personal fees from Daiichi Sankyo, personal fees from BMS, personal fees from MSD, grants, personal fees and other from Medtronic, personal fees from Pfizer, personal fees from Merit Medical, grants, personal fees and other from St Jude MEdical /ABBOTT, personal fees from Bayer, personal fees from Mylan, personal fees from Pro Med CS, personal fees from Merck, outside the submitted work. KHK reports grants and minor personal fees from Medtronic, Biosense Webster and Impulse Dynamics. KW reports grants from AF-Net, during the conduct of the study; grants from Biotronik, personal fees from Biotronik, personal fees from Boston Scientific, from Resmed, from Novartis, outside the submitted work. LM is shareholder of Galgo Medical, S.L. LMH reports grants from Abbott, grants from Abiomed, grants from Amgen, grants from Astra Zeneca, grants from Bayer, grants from Biosense Webster, grants from Biotronik, grants from Boston Scientific, grants from Bracco, grants from B. Braun, grants and personal fees from Daiichi-Sankyo, grants from Edwards Lifesciences, grants and personal fees from Medtronic, grants from MicroPort, grants from Novartis, grants from Vascular Medical, grants from Zoll, outside the submitted work. LS reports leadership or fiduciary role in other board, society, committee or advocacy group, paid or unpaid for Advisory Board for Cardiology for Polish Ministry of Health. PK reports grants and non-financial support from BMBF (German Ministry of Education and Research), grants from Sanofi, grants from Abbott, grants and non-financial support from EHRA (European Heart Rhythm Association), grants from German Heart Foundation, grants from DZHK (German Center for Cardiovascular Research), during the conduct of the study; grants from European Union, grants from British Heart Foundation, grants from Leducq Foundation, grants from Medical Research Council (UK), non-financial support from German Centre for Heart Research, outside the submitted work; In addition, Dr. Kirchhof has a patent Atrial Fibrillation Therapy WO 2015140571 issued to University of Birmingham, and a patent Markers for Atrial Fibrillation WO 2016012783 issued to University of Birmingham. PV reports personal fees from Servier, personal fees from Hygeia Hospitals Group, personal fees from Dean Medicus LTD, personal fees from Bayer, personal fees from European Society of Cardiology, personal fees from Menarini, outside the submitted work. SW repots grants and personal fees from Boston Scientific, personal fees from Boehringer Ingelheim, grants and personals fees from Abbott, personal fees from Bristol Myers Squibb, personal fees from Byer Vital, oersonal fees from Daiichi Sankyo, outside the submitted work. LE, AE and ST have nothing to disclose.

## Data availability

We will share all data that support published results of the trial. Data will be made available as required for approved analyses. Requests can be made to east@af-net.eu and will be reviewed by AFNET.

## Supplementary Material

euab200_Supplementary_Data
