# **Corrigendum to:** Anticoagulation, therapy of concomitant conditions, and early rhythm control therapy: a detailed analysis of treatment patterns in the EAST-AFNET 4 trial

**DOI:** 10.1093/europace/euab277

**Published:** 2021-12-13

**Authors:** Andreas Metzner, Anna Suling, Axel Brandes, Günter Breithardt, A John Camm, Harry J G M Crijns, Lars Eckardt, Arif Elvan, Andreas Goette, Laurent M Haegeli, Hein Heidbuchel, Josef Kautzner, Karl-Heinz Kuck, Luis Mont, G Andre Ng, Lukasz Szumowski, Sakis Themistoclakis, Isabelle C van Gelder, Panos Vardas, Karl Wegscheider, Stephan Willems, Paulus Kirchhof

*Europace* 2021; https://doi.org/10.1093/europace/euab200

In the originally published version of this manuscript, an author’s name was incorrect. The author’s name should read “G. Andre Ng” instead of “A. Andre Ng”. This error has been corrected.

