# Supplementary material for: Anticoagulation, therapy of concomitant conditions, and early rhythm control therapy: a detailed analysis of treatment patterns in the EAST - AFNET 4 trial
Source: Europace. 2021 Sep 2;24(4):552–64. doi: 10.1093/europace/euab200 (PMC8982435; doi:10.1093/europace/euab200)
Supplement: euab200_Supplementary_Data [file euab200_supplementary_data.docx]

**Components of AF management and early rhythm control in patients with atrial fibrillation: a detailed analysis of the EAST-AFNET 4 dataset.**

Metzner A^1, 2^, Suling A^3^, Brandes A^4, 5^, Breithardt G^6, 7^, Camm AJ^8^, Crijns HJGM^9^, Eckardt L^6,7^, Elvan A^10^, Goette A^6, 11,12^, Haegeli LM^13, 14^, Heidbuchel H^15^, Kautzner J^16^, Kuck KH^2, 6, 17^, Mont L^18, 19^, Ng GA^20^, Szumowski L^21^, Themistoclakis S^22^, van Gelder IC^23^, Vardas P^24^, Wegscheider K^2,3,6^, Willems S^2, 6, 25^, Kirchhof P^1, 2, 6, 26^

^1^ Department of Cardiology, University Heart and Vascular Center, University Medical Center Hamburg–Eppendorf, Germany

^2^German Center of Cardiovascular Research, Partner Site Hamburg/Lübeck/Kiel, Germany

^3^Institute of Medical Biometry and Epidemiology, University Medical Center Hamburg–Eppendorf, Germany

^4^Department of Cardiology, Odense University Hospital, Denmark

^5^Department of Clinical Research, University of Southern Denmark, Odense, Denmark

^6^Atrial Fibrillation Network (AFNET), Münster, Germany

^7^Department of Cardiology II (Electrophysiology), University Hospital Münster, Germany

^8^Cardiology Clinical Academic Group, Molecular and Clinical Sciences Research Institute, St. George’s University of London, UK

^9^Department of Cardiology, Maastricht University Medical Center and Cardiovascular Research Institute Maastricht, Netherlands

^10^Isala Hospital and Diagram Research, Zwolle, The Netherlands

^11^St. Vincenz Hospital, Paderborn, Germany

^12^Working Group of Molecular Electrophysiology, University Hospital Magdeburg, Germany

^13^University Hospital Zurich, Zurich, Switzerland

^14^Division of Cardiology, Medical University Department, Kantonsspital Aarau, Switzerland

^15^University Hospital Antwerp and Antwerp University, Antwerp, Belgium

^16^Institute for Clinical and Experimental Medicine, Prague, Czech Republic

^17^LANS Cardio, Hamburg, Germany

^18^University of Barcelona and Institut de Recerca Biomèdica, August Pi-Sunyer, Barcelona, Spain

^19^Centro Investigación Biomedica en Red Cardiovascular, Madrid, Spain

^20^Department of Cardiovascular Sciences, University of Leicester, National Institute for Health Research Leicester Biomedical Research Centre, Glenfield Hospital, Leicester, UK

^21^Arrhythmia Center of the National Institute of Cardiology; Medical Division of Cardinal Stefan Wyszynski University in Warsaw. Warsaw, Poland

^22^Department of Cardiology, Ospedale dell’Angelo, Venice, Italy

^23^University of Groningen, University Medical Center Groningen, Groningen, Netherlands

^24^Heart Sector, Hygeia Hospitals Group, Athens, Greece

^25^Department of Cardiology, Asklepios Klinik St. Georg (S.W.), Hamburg, Germany

^26^Cardiovascular Sciences, University of Birmingham, Birmingham, UK

**Correspondence to**

Paulus Kirchhof, MD

Department of Cardiology

University Heart and Vascular Center UKE Hamburg

Martinistraße 52

20246 Hamburg, Germany

Email [p.kirchhof@uke.de](mailto:p.kirchhof@uke.de)

Phone +49 40 741053824

**Abstract** (238 words)

**Background** Treatment patterns were compared between randomised groups in EAST-AFNET 4 to assess whether differences in antiocoagulation, therapy of concomitant diseases, or intensity of care can explain the clinical benefit achieved with early rhythm control in EAST – AFNET 4.

**Methods** Cardiovascular treatment patterns and number of visits were compared between randomised groups in EAST – AFNET 4.

**Results.** Oral anticoagulation was used in >90% of patients during follow-up without differences between randomized groups. There were no differences in treatment of concomitant conditions between groups. The type of rhythm control varied by country and center. Over time, antiarrhythmic drugs were given to 1171/1395 (84%) patients in early therapy, and to 202/1394 (14%) in usual care. AF ablation was performed in 340/1395 (24%) patients randomized to early therapy, and in 168/1394 (12%) patients randomized to usual care. 97% of rhythm control therapies were within class I and class III recommendations of AF guidelines. Patients randomised to early therapy transmitted 297,166 telemetric ECGs to a core lab. 97,978 abnormal ECGs were sent to study sites. The resulting difference between study visits was low (0.06 visits/patient/year), with slightly more visits in early therapy (usual care 0.39 visits/patient/year; early rhythm control 0.45 visits/patient/year, p<0.001), mainly due to visits for symptomatic AF recurrences or recurrent AF on telemetric ECGs.

**Conclusion.** The clinical benefit of early, systematic rhythm control therapy was achieved using variable treatment patterns of antiarrhythmic drugs and AF ablation, applied within guideline recommendations.

**What´s new.** This analysis provides critical new and detailed information, showing the following:

1. Early rhythm control therapy was delivered on top of high oral anticoagulation rates and high use of rate control in both randomized groups.
2. There were no relevant differences in other cardiovascular treatments that could explain the outcome of the trial.
3. Early rhythm control therapy was achieved with a very low number of study visits. Telemetric ECG monitoring with over 300,000 transmitted ECG devices only resulted in ca 150 extra visits over 5 years.
4. Confirming other recent trials, the analysis confirms the safety of rhythm control therapy.
5. The clinical benefit of early, systematic rhythm control therapy was achieved using variable treatment patterns of antiarrhythmic drugs and AF ablation, applied within guideline recommendations. These patterns can be followed to implement early rhythm control therapy for all patients with recently diagnosed atrial fibrillation and concomitant conditions.

**Introduction**

Optimal management of patients with atrial fibrillation (AF) includes anticoagulation, rate control therapy, and therapy of concomitant cardiovascular conditions, which may be supplemented by rhythm control therapy in patients who remain symptomatic on optimal rate control according to current guidelines^1, 2^. Even on optimal therapy, patients with AF remain at high risk of cardiovascular death (1-2%/year) ^3-6^, worsening of heart failure (3.5% of patients hospitalised for heart failure/year ^4, 5, 7^) and stroke despite appropriate anticoagulation (1%/year ^8^). Indeed, 5% of well-managed AF patients experience these severe complications per year. ^6, 9^

The EAST – AFNET 4 trial demonstrated that systematic, early initiation of rhythm control therapy results in a 21% relative risk reduction in a composite of cardiovascular death, stroke, and hospitalization for heart failure or acute coronary syndrome in a population of patients with recently diagnosed AF and concomitant cardiovascular conditions. ^9, 10^ The clinical benefit was achieved with equal overall safety, including fewer strokes, numerically lower mortality and more serious adverse events related to rhythm control therapy in patients randomized to early rhythm control. To provide context for this finding, and to enable delivery of early rhythm control therapy in clinical practice, the treatment patterns used in EAST – AFNET 4 need to be known in detail. Furthermore, unintended differences in the delivery of other components of AF therapy such as anticoagulation, therapy of concomitant cardiovascular conditions, or more intensive contacts with the study sites could have influenced the outcome of the study.

To increase understanding of the trial results and to enable their clinical implementation ^9, 11^, treatment patterns were compared between randomized groups in the EAST – AFNET 4 trial population including anticoagulation, therapy of concomitant cardiovascular conditions, rate control therapy, study visits, and rhythm control therapy.

**Methods**

This is a comparison of the treatment components between randomized groups in the EAST – AFNET 4 trial, and of the factors associated with specific therapies in the EAST – AFNET 4 dataset. The design of the EAST – AFNET 4 trial, the methods of analysis, and the main results have been published. ^6, 9^ The current analysis was performed on the final, locked database of the trial. Analyses included treatments at discharge from the randomization visit, at one year of follow-up, and at two years of follow-up. Descriptive data on the use of different therapies, including anticoagulation, therapy of concomitant cardiovascular conditions, rate control, and rhythm control therapy as well as the number of visits were summarized. In addition, therapies were classified as guideline-mandated based on the class I recommendations of ESC practice guidelines in use at the time. ^2, 12, 13^ Treatment patterns were described and analyzed for differences between randomized groups, clinical characteristics, and center and country effects. Treatment changes over time were analyzed and compared between randomized groups.

Continuous variables are reported as mean and standard deviation and categorical variables are presented as frequencies and percentages. For visualization, bar plots, box plots, and Aalen-Johansen cumulative incidence curves, accounting for the competing risk of death, were used. To determine the relation between administered rhythm control (AAD, ablation, or none), anticoagulation therapy and potential factors (e.g., age, gender, country), we used mixed logistic regression models adjusted for the random effect of center. Results are presented as Odds Ratios (OR) together with 95%-confidence intervals (CI).

Mixed logistic regression models were also used to assess differences between treatment groups in the cardiovascular therapies, participation of main follow-up visits and apparent violations of class I recommendations (or Fisher’s exact test if the mixed logistic regression model was not applicable). Mixed Poisson and mixed linear regression models were used to assess differences in the number of visits per patient and the number of visits per patient per year, respectively. Both model types were unadjusted and included a random term for the center effect. All analyses were performed using STATA 16.1 (StataCorp. 2019) and R 4.0.2 (R Core Team 2020). The authors had access to the entire, locked database of the trial and vouch for the fidelity of the data and their analyses.

**Results**

Between July 2011 and December 2016, 135 sites in 11 countries randomized 2789 patients to the EAST – AFNET 4 trial. Over half of the sites participating in EAST were smaller sites without on-site ablation facilities who cooperated with ablation centers. A total of 1752 patients (63%) were randomised in sites without on-site ablation facilities (called D-sites, **Suppl. Table 1**), the remaining 1037 patients in sites performing AF ablation on-site (called A-sites). University hospitals randomised 579 (21%) patients, other hospitals 1276 (46%) patients, and office-based cardiologists 934 (33%) patients.

Over 90% of patients received guideline-mandated oral anticoagulation throughout the follow-up without differences between randomised groups (**Table 1, Suppl. Table 2, Figure 1**). In a multivariate analysis, anticoagulation therapy at any time was influenced by patient’s age (OR 1.64, 95%-CI [1.36;1.98]; p<0.001), gender (male vs. female OR 1.42, 95%-CI [1.42, 95%-CI [1.00; 2.01]; p=0.048) and AF pattern (persistent or long-standing persistent vs. first episode or paroxysmal OR 3.38, 95%-CI [1.81;6.31]; p<0.001), without differences between randomized groups (p=0.912). The use of novel oral anticoagulants (NOACs) was high (>54% at baseline in both groups) with a slight further increase during follow-up.

Therapy of concomitant cardiovascular conditions appeared well balanced, with about 70% of patients receiving inhibitors of the renin-angiotensin-aldosterone system. Blood pressure was not different between randomized groups throughout follow-up (**Table 1,** **Suppl. Table 2, Figure 2**).

Rate control therapy was used in most patients. Overall, 1088/1389 (78.3%) patients randomised to early rhythm control therapy received beta-blockers, verapamil or diltiazem, or digitalis glycosides at discharge from the baseline visit, and 1235/1393 (88.7%) patients randomised to usual care. When the use of antiarrhythmic drugs with rate controlling properties (amiodarone, dronedarone, propafenone, or sotalol) was included in the analysis, the difference in rate control was much less pronounced (**Table 1, Figure 3**). The use of rate control decreased during follow-up in both groups, more in patients randomized to early rhythm control.

In-person visits were infrequent during the median FU of 5.1 years per patient due to the study design. ^6^ Patients randomised to early rhythm control therapy underwent 2974 in-person visits (2.13/patient, 0.45 visits/patient/year) including 249 visits triggered by detection of recurrent AF calling for an adjustment of rhythm control therapy (so-called triggered visits) ^6^, slightly more than the 2710 visits (1.94/patient, 0.39 visits/patient/year) including 93 triggered visits in patients randomized to usual care (**Table 2**, **Figure 4A**). The increase in site visits seen in patients randomized to early therapy was mainly driven by triggered visits to adjust rhythm control therapy (**Figure 4B**). Patients randomised to early therapy transmitted 297,166 telemetric, thirty-second ECG recordings to a core lab. Of these, 97,978 were judged as abnormal and sent to study sites for review and to decide on clinical consequences. Only a small number of abnormal telemetric ECGs led to clinical actions: Of the 249 triggered visits performed in patients randomised to early rhythm control, approximately 150 were due to abnormal telemetric ECGs.

Of 2789 patients, 508 patients (18.2%) received an ablation at any time, with 340/1395 (24%) patients randomized to early therapy receiving ablation. Antiarrhythmic drug therapy was given to 1373 (49.2%) patients, including 1171/1395 (84%) of those randomized to early therapy. A total of 1208/2789 (43.3%) were managed without ablation or antiarrhythmic drug therapy throughout the trial (usual care: 1079/1394 (77%)). Almost all patients (>97% of those receiving rhythm control therapy) received rhythm control therapy aligned with the class I recommendations in guidelines (**Table 3**). Some centers preferentially used AF ablation for rhythm control management, reflecting access to therapy and preferences by the local study teams. Others preferentially used flecainide, propafenone, dronedarone, or other amiodarone as initial rhythm control therapy in the majority of their patients. Adjustments to rhythm control therapy were relatively common in the first year after randomization, predominantly in patients randomized to early rhythm control (**Figure 5A**). Many ablations were performed immediately following randomization to early rhythm control. Thereafter, the number of patients treated by ablation increased steadily in both randomized groups (**Figure 5B**). At two years, 270/1395 (19.4%) patients randomized to early rhythm control therapy had undergone AF ablation, while 97/1394 (6.9%) patients randomized to usual care had undergone ablation. This corresponded to 26.7% of patients still in follow-up at 2 years. The decision to manage a patient without rhythm control therapy was almost exclusively explained by randomized group without any relevant other effects (OR early treatment vs. usual care 0.02, 95%-CI [0.02;0.03]; p<0.001), **Figure 6A, Suppl. Table 4**). The initial choice of the type of rhythm control therapy varied by center (**Figure 6B**). AF ablation was more likely given to patients randomized to early treatment, patients recruited in an A-site or in another country than Spain, Italy or Poland, younger patients, those without diabetes mellitus, and patients included with first diagnosed or paroxysmal AF (**Figure 6 and Suppl. Table 3**). If patients randomized to usual care remained symptomatic despite optimal rate control, the trial protocol called for rhythm control initiation by means of antiarrhythmic drugs or ablation. The high proportion of patients without AF-related symptoms (EHRA I) in both randomized groups at two years substantiates the adequate, protocol-conform use of rhythm control to improve AF-related symptoms in the usual care arm.

**Discussion**

**Main findings.** This in-depth analysis of the therapies given to patients participating in the EAST – AFNET 4 trial produced three major results.

1. A strategy of systematic and early rhythm control therapy achieved clinical benefit when added to evidence-based anticoagulation and rate control therapy.
2. There were no relevant differences in other cardiovascular treatments that could explain the outcome of the trial.
3. EAST – AFNET 4 implemented early rhythm control without many additional visits: On average, each patient was seen 1.94 (usual care) and 2.13 (early therapy) times by the study center during the follow-up of approximately 5 years.
4. Early, systematic rhythm control was achieved using a combination of antiarrhythmic drugs and AF ablation. Early rhythm control treatment patterns varied by site and country within guideline recommendations, outlining a range of ways to provide early rhythm control therapy to patients with atrial fibrillation.

**Anticoagulation.** Evidence-based anticoagulation use was high (>90% throughout follow-up) without differences between randomized groups. Approximately half of the patients were treated with NOACs at discharge from randomization, increasing slightly at two years (**Table 1**), comparable to concomitant and more recent large European observational data sets. ^14, 15^ The adequate, continued use of anticoagulants and the high therapy adherence can explain the low stroke rate observed in EAST – AFNET 4 ^9^, consistent with reports from large anticoagulation trials, and different from the AFFIRM trial. ^4, 16^

**Concomitant cardiovascular conditions** were treated without differences between randomised groups. Blood pressure, an important surrogate outcome associated with stroke and other cardiovascular events, was not different between randomized groups. There were 4-6% more patients randomized to early therapy who received statins. While this difference was significant, and can contribute to a reduction in acute coronary syndrome, stroke, and even cardiovascular death, it is very small. In view of the balanced distribution of therapies for other cardiovascular comorbidities, the lack of differences in blood pressure between randomized groups, and in view of the long-term outcomes of RACE-3 ^17^, where a randomized intervention with high use of statins, MRAs, and nurse-led care did not improve five-year outcomes (neither for recurrent AF nor for MACCE, recently presented at EHRA 2021), it is unlikely that undetected differences in this treatment domain can explain the differences in outcomes observed in EAST – AFNET 4.

**Rate control therapy** was given to the vast majority of patients in EAST – AFNET 4, in line with current guidelines (**Table 1**). Digoxin was used in a very small number of patients, and almost entirely as second-line therapy on top of beta-blockers, following current recommendations and trial results.^18^ Whether this remains best practice in patients with AF and heart failure remains to be tested in light of the recently published RATE-AF trial. ^19^

**Number of visits.** The number of study visits was low in both study arms, but slightly and significantly higher in patients randomized to early therapy (usual care 0.39 visits/patient/year, early rhythm control 0.45 visits/patient/year, p<0.001). As can be appreciated in Figure 1, most of these visits occurred early after randomization, most likely to adjust rhythm control therapy. The number of extra visits induced by telemetric ECG monitoring is lower than expected at the start of the trial. ^6^ As the results of abnormal telemetric ECG recordings were only revealed to study sites ^6^ , this small increase in study visits will capture almost all additional visits induced by telemetric ECG monitoring (**Table 2, Figure 4A).** Together with the reported finding that there was no difference in nights spent in hospital between groups ^8^, these data demonstrate that early therapy was delivered with few added visits, and that differences in the intensity of care between groups cannot explain the observed effects of early therapy on cardiovascular death, stroke, and hospitalizations for heart failure or acute coronary syndrome. While delivery of care in a controlled trial will differ from routine clinical care, the excellent delivery of all domains of AF care in the EAST –AFNET 4 center networks with few planned or unplanned visits may provide exemplars for the delivery of holistic, integrated, cost-effective care for patients with AF.

**Rhythm control therapy** was well aligned with guidelines, with >97% of control therapies following accepted class I recommendations (**Table 3**). ^2, 12^ Early rhythm control was initially delivered as antiarrhythmic drug therapy in most patients, and ¾ of patients were treated without AF ablation throughout the trial. AF ablation was used in ca ¼ of patients randomized to early therapy **Figure 5B**), illustrating the importance of this treatment modality in the trial. As expected for early rhythm control, the difference between the use of AF ablation was most marked in the first few months after randomization (**Figure 5B**). In patients randomized to usual care, rhythm control was used in 15% of patients at two years, very similar to general AF registries reporting rhythm control^15, 20, 21^ and at a rate anticipated in the design of the trial ^5^. In addition to randmisation to early rhythm control, the use of AF ablation was associated with enrolment at an A-site, younger age, no diabetes mellitus, and with first diagnosed or paroxysmal AF, **Figure 6A**). Furthermore, there were regional differences in the use of AF ablation, probably reflecting the access to AF ablation at the time of enrolment into the trial (2011 – 2016).Furthermore, regional differences in the competence and practice of antiarrhythmic drug therapy probably drove these differences.

Sinus rhythm rates were higher on early rhythm control in EAST – AFNET 4 (80% at two years^8^) than in AFFIRM^22^ or AF-CHF^23^, illustrating the effectiveness of the early therapy strategy. The high rate of sinus rhythm in the early treatment arm might be explained by the modern rhythm control therapy patterns including safe use of sodium channel blockers, treatment with dronedarone, and AF ablation. These components of rhythm control therapy were not available at the time of AFFIRM and only rarely use in AF-CHF. The early timing of rhythm control therapy can furthermore explain the high rate of sinus rhythm.^24^

**Treatment patterns used to deliver early rhythm control therapy.** EAST – AFNET 4 was a strategy trial. The vast majority of the rhythm control therapy options used in EAST – AFNET 4 (ca 97%, **Table 3**) are supported by AF treatment guidelines ^2, 12^ and led to few safety events due to antiarrhythmic drug or AF ablation ^3, 25^. EAST – AFNET 4 enrolled patients from 2011 to 2016. While the use of AF ablation was high for the practice at the time, it seems likely that contemporary rhythm control therapy may make more use of AF ablation in light of recent data illustrating its safety ^3, 25^, improvement in quality of life ^26, 27^, and effectiveness in maintaining sinus rhythm. ^28, 29^

A high degree of center-based variation was found in the initial selection of rhythm control therapy. This is in keeping with reports from the Veterans Administrations database where center-based effects were a key determinant of the choice of antiarrhythmic drug. ^25^ Possible drivers of these differences are local experience, protocols, access to therapy options, reimbursement, and others. ^30^ The clinical benefit of early rhythm control was not affected by type of center, underpinning that different treatment patterns can be used to achieve early rhythm control. Important for the interpretation of the trial is that all centers had access to AF ablation performed in experienced centers.

The current analysis emphasises the relevance of AF ablation for safe and effective rhythm control therapy, used in 24% of patients randomized to early rhythm control therapy, but also the effectiveness of antiarrhythmic drugs when initiated early, sufficient in around 75% of patients to deliver early rhythm control therapy. It is likely that sinus rhythm, lack of documented or symptomatic AF recurrences, failure of rhythm control, and patient preferences were the drivers of discontinuation of rhythm control therapy during the course of the study in circa 35% of patients randomized to early rhythm control at 2 years (**Figure 5A**).

**Limitations.** While the EAST – AFNET 4 trial enrolled almost 3000 patients in 11 European countries with different health care systems, actively enrolling in sites with and without on-site AF ablation, small cardiology practices and large tertiary care centers, reflecting different treatment patterns and cultures, there may be further, different rhythm control treatment patterns with equal effectiveness. It is likely that different patterns and potentially different outcomes could arise from contemporary delivery of rhythm control, e.g., more AF ablations. It is unclear whether differences in therapy choices had an effect on outcomes. This requires complex modelling that is beyond the scope of this analysis.

**Conclusions.** Different patterns of early rhythm control therapy resulted in lower rates of cardiovascular death, stroke, and hospitalizations for heart failure or acute coronary syndrome when added to a comprehensive management of AF including anticoagulation, therapy of concomitant cardiovascular conditions, and rate control therapy. There were no differences between randomised groups other than the study intervention that could explain the difference in clinical outcomes. Early rhythm control was delivered using different treatment patterns, providing a range of choices how to deliver early rhythm control therapy to achieve clinical benefit in patients with AF.

**Data availability**: We will share all data that support published results of the trial. Data will be made available as required for approved analyses. Requests can be made to east@af-net.eu and will be reviewed by AFNET.

**Conflicts of interest:**

Breithardt and Kirchhof: grants or support for AFNET from DZHK, EHRA, DHS, Abott Laboratories, Sanofi.

Suling and Wegscheider: grant from AFNET for statistical analysis

**Funding**: Funded by AFNET, DZHK, EHRA, DHS, Abbott Laboratories, Sanofi; EAST-AFNET 4 ISRCTN number, ISRCTN04708680; ClinicalTrials.gov number, NCT01288352; EudraCT number, 2010 -021258-20.

**References**

[1] Hindricks G, Potpara T, Dagres N, Arbelo E, Bax JJ, Blomstrom-Lundqvist C, et al. 2020 ESC Guidelines for the diagnosis and management of atrial fibrillation developed in collaboration with the European Association for Cardio-Thoracic Surgery (EACTS). *Eur Heart J* 2021; **42**: 373-498.

[2] January CT, Wann LS, Calkins H, Chen LY, Cigarroa JE, Cleveland JC, Jr., et al. 2019 AHA/ACC/HRS Focused Update of the 2014 AHA/ACC/HRS Guideline for the Management of Patients With Atrial Fibrillation: A Report of the American College of Cardiology/American Heart Association Task Force on Clinical Practice Guidelines and the Heart Rhythm Society in Collaboration With the Society of Thoracic Surgeons. *Circulation* 2019; **140**: e125-e151.

[3] Packer DL, Mark DB, Robb RA, Monahan KH, Bahnson TD, Poole JE, et al. Effect of Catheter Ablation vs Antiarrhythmic Drug Therapy on Mortality, Stroke, Bleeding, and Cardiac Arrest Among Patients With Atrial Fibrillation: The CABANA Randomized Clinical Trial. *JAMA* 2019; **321**: 1261-1274.

[4] Marijon E, Le Heuzey JY, Connolly S, Yang S, Pogue J, Brueckmann M, et al. Causes of death and influencing factors in patients with atrial fibrillation: a competing-risk analysis from the randomized evaluation of long-term anticoagulant therapy study. *Circulation* 2013; **128**: 2192-2201.

[5] Kotecha D, Breithardt G, Camm AJ, Lip GYH, Schotten U, Ahlsson A, et al. Integrating new approaches to atrial fibrillation management: the 6th AFNET/EHRA Consensus Conference. *Europace* 2018; **20**: 395-407.

[6] Kirchhof P, Breithardt G, Camm AJ, Crijns HJ, Kuck KH, Vardas P, et al. Improving outcomes in patients with atrial fibrillation: rationale and design of the Early treatment of Atrial fibrillation for Stroke prevention Trial. *Am Heart J* 2013; **166**: 442-448.

[7] Willems S, Meyer C, de Bono J, Brandes A, Eckardt L, Elvan A, et al. Cabins, castles, and constant hearts: rhythm control therapy in patients with atrial fibrillation. *Eur Heart J* 2019; **40**: 3793-3799c.

[8] Camm AJ, Amarenco P, Haas S, Hess S, Kirchhof P, Kuhls S, et al. XANTUS: a real-world, prospective, observational study of patients treated with rivaroxaban for stroke prevention in atrial fibrillation. *Eur Heart J* 2016; **37**: 1145-1153.

[9] Kirchhof P, Camm AJ, Goette A, Brandes A, Eckardt L, Elvan A, et al. Early Rhythm-Control Therapy in Patients with Atrial Fibrillation. *N Engl J Med* 2020; **383**: 1305-1316.

[10] Gillis AM, Wilton SB. Rhythm Control of Atrial Fibrillation: The Earlier the Better? *Circulation* 2021; **143**: 1639-1641.

[11] Bunch TJ, Steinberg BA. Revisiting Rate versus Rhythm Control in Atrial Fibrillation - Timing Matters. *N Engl J Med* 2020; **383**: 1383-1384.

[12] Kirchhof P, Benussi S, Kotecha D, Ahlsson A, Atar D, Casadei B, et al. 2016 ESC Guidelines for the management of atrial fibrillation developed in collaboration with EACTS. *Eur Heart J* 2016; **37**: 2893-2962.

[13] Camm AJ, Lip GY, De Caterina R, Savelieva I, Atar D, Hohnloser SH, et al. 2012 focused update of the ESC Guidelines for the management of atrial fibrillation: an update of the 2010 ESC Guidelines for the management of atrial fibrillation. Developed with the special contribution of the European Heart Rhythm Association. *Eur Heart J* 2012; **33**: 2719-2747.

[14] Le Heuzey JY, Ammentorp B, Darius H, De Caterina R, Schilling RJ, Schmitt J, et al. Differences among western European countries in anticoagulation management of atrial fibrillation. Data from the PREFER IN AF registry. *Thromb Haemost* 2014; **111**: 833-841.

[15] Potpara TS, Lip GYH, Dagres N, Crijns H, Boriani G, Kirchhof P, et al. Cohort profile The ESC EURObservational Research Programme Atrial Fibrillation III (AF III) Registry. *Eur Heart J Qual Care Clin Outcomes* 2020.

[16] Ruff CT, Giugliano RP, Braunwald E, Hoffman EB, Deenadayalu N, Ezekowitz MD, et al. Comparison of the efficacy and safety of new oral anticoagulants with warfarin in patients with atrial fibrillation: a meta-analysis of randomised trials. *Lancet* 2014; **383**: 955-962.

[17] Rienstra M. Targeted therapy of underlying conditions in patients with persistent atrial fibrillation and mild to moderat stable heart failure: long-term outcome of the RACE 3 Trial

[18] Van Gelder IC, Groenveld HF, Crijns HJ, Tuininga YS, Tijssen JG, Alings AM, et al. Lenient versus strict rate control in patients with atrial fibrillation. *N Engl J Med* 2010; **362**: 1363-1373.

[19] Kotecha D, Bunting KV, Gill SK, Mehta S, Stanbury M, Jones JC, et al. Effect of Digoxin vs Bisoprolol for Heart Rate Control in Atrial Fibrillation on Patient-Reported Quality of Life: The RATE-AF Randomized Clinical Trial. *JAMA* 2020; **324**: 2497-2508.

[20] Kirchhof P, Ammentorp B, Darius H, De Caterina R, Le Heuzey JY, Schilling RJ, et al. Management of atrial fibrillation in seven European countries after the publication of the 2010 ESC Guidelines on atrial fibrillation: primary results of the PREvention oF thromboemolic events--European Registry in Atrial Fibrillation (PREFER in AF). *Europace* 2014; **16**: 6-14.

[21] Noheria A, Shrader P, Piccini JP, Fonarow GC, Kowey PR, Mahaffey KW, et al. Rhythm Control Versus Rate Control and Clinical Outcomes in Patients With Atrial Fibrillation: Results From the ORBIT-AF Registry. *JACC Clin Electrophysiol* 2016; **2**: 221-229.

[22] Wyse DG, Waldo AL, DiMarco JP, Domanski MJ, Rosenberg Y, Schron EB, et al. A comparison of rate control and rhythm control in patients with atrial fibrillation. *N Engl J Med* 2002; **347**: 1825-1833.

[23] Roy D, Talajic M, Nattel S, Wyse DG, Dorian P, Lee KL, et al. Rhythm control versus rate control for atrial fibrillation and heart failure. *N Engl J Med* 2008; **358**: 2667-2677.

[24] Nattel S, Guasch E, Savelieva I, Cosio FG, Valverde I, Halperin JL, et al. Early management of atrial fibrillation to prevent cardiovascular complications. *Eur Heart J* 2014; **35**: 1448-1456.

[25] Cosedis Nielsen J, Johannessen A, Raatikainen P, Hindricks G, Walfridsson H, Kongstad O, et al. Radiofrequency ablation as initial therapy in paroxysmal atrial fibrillation. *N Engl J Med* 2012; **367**: 1587-1595.

[26] Mark DB, Anstrom KJ, Sheng S, Piccini JP, Baloch KN, Monahan KH, et al. Effect of Catheter Ablation vs Medical Therapy on Quality of Life Among Patients With Atrial Fibrillation: The CABANA Randomized Clinical Trial. *JAMA* 2019; **321**: 1275-1285.

[27] Blomstrom-Lundqvist C, Gizurarson S, Schwieler J, Jensen SM, Bergfeldt L, Kenneback G, et al. Effect of Catheter Ablation vs Antiarrhythmic Medication on Quality of Life in Patients With Atrial Fibrillation: The CAPTAF Randomized Clinical Trial. *JAMA* 2019; **321**: 1059-1068.

[28] Andrade JG, Wells GA, Deyell MW, Bennett M, Essebag V, Champagne J, et al. Cryoablation or Drug Therapy for Initial Treatment of Atrial Fibrillation. *N Engl J Med* 2021; **384**: 305-315.

[29] Wazni OM, Dandamudi G, Sood N, Hoyt R, Tyler J, Durrani S, et al. Cryoballoon Ablation as Initial Therapy for Atrial Fibrillation. *N Engl J Med* 2021; **384**: 316-324.

[30] Glorioso TJ, Grunwald GK, Ho PM, Maddox TM. Reference effect measures for quantifying, comparing and visualizing variation from random and fixed effects in non-normal multilevel models, with applications to site variation in medical procedure use and outcomes. *BMC Med Res Methodol* 2018; **18**: 74.

**Figures and Tables**

**Table 1:** Cardiovascular therapies given to patients in the EAST – AFNET 4 trial at discharge from the baseline visit, at 12 months FU, and at 24 months follow-up. All patient numbers are given split by randomized group and in total. Proportions indicate proportions of patients receiving each therapy at each time point as a fraction of the totality of patients still in follow-up and with available medication information at that time point. Anticoagulation, therapy with heart failure and antihypertensive drugs, antidiabetic therapy, and rate control therapy were used in most patients. *Antiarrhythmic drugs with rate controlling properties are amiodarone, dronedarone, propafenone, and sotalol. p values resulting from mixed logistic regression with center as random effect.

|  | **Randomized group** | | |  | |  | | | |
| --- | --- | --- | --- | --- | --- | --- | --- | --- | --- |
|  | | **Early rhythm control (N=1395)** | **Usual care (N=1394)** | | **Total (N=2789)** | | **p-value** |  |  |
| **Patients receiving oral anticoagulation** | | | | | | |  | |  |
| Anticoagulation (discharge from baseline) | | 1267/1389 (91.2%) | 1250/1393 (89.7%) | | 2517/2782 (90.5%) | | 0.149 |  |  |
| NOACs (discharge from BL) | | 800/1389 (57.6%) | 763/1393 (54.8%) | | 1563/2782 (56.2%) | | 0.103 |  |  |
| Vitamin K antagonists (discharge from BL) | | 467/1389 (33.6%) | 490/1393 (35.2%) | | 957/2782 (34.4%) | | 0.397 |  |  |
| Anticoagulation (12 months FU) | | 1087/1230 (88.4%) | 1121/1241 (90.3%) | | 2208/2471 (89.4%) | | 0.111 |  |  |
| NOACs (12 months FU) | | 713/1230 (58.0%) | 704/1241 (56.7%) | | 1417/2471 (57.3%) | | 0.657 |  |  |
| Vitamin K antagonists (12 months FU) | | 376/1230 (30.6%) | 421/1241 (33.9%) | | 797/2471 (32.3%) | | 0.100 |  |  |
| Anticoagulation (24 months FU) | | 1020/1159 (88.0%) | 1065/1171 (90.9%) | | 2085/2330 (89.5%) | | 0.021 |  |  |
| NOACs (24 months FU) | | 690/1159 (59.5%) | 699/1171 (59.7%) | | 1389/2330 (59.6%) | | 0.774 |  |  |
| Vitamin K antagonists (24 months FU) | | 330/1159 (28.5%) | 366/1171 (31.3%) | | 696/2330 (29.9%) | | 0.202 |  |  |
| **Patients receiving rate control therapy (beta adrenoreceptor blocker, verapamil, diltiazem, or digitalis glycosides)** | | | | | | |  | |  |
| Rate control (discharge from BL) | | 1088/1389 (78.3%) | 1235/1393 (88.7%) | | 2323/2782 (83.5%) | | <0.001 |  |  |
| Rate control (12 months FU) | | 883/1230 (71.8%) | 1055/1241 (85.0%) | | 1938/2471 (78.4%) | | <0.001 |  |  |
| Rate control (24 months FU) | | 799/1159 (68.9%) | 986/1171 (84.2%) | | 1785/2330 (76.6%) | | <0.001 |  |  |
| **Patients receiving any rate controlling medication (b adrenoreceptor blocker, verapamil, diltiazem, digitalis glycosides, or antiarrhythmic drugs with rate controlling properties*)** | | | | | | |  | |  |
| Patients receiving any rate controlling medication (discharge from BL) | | 1259/1389 (90.6%) | 1250/1393 (89.7%) | | 2509/2782 (90.2%) | | 0.382 |  |  |
| Patients receiving any rate controlling medication (12 months FU) | | 1065/1230 (86.6%) | 1084/1241 (87.3%) | | 2149/2471 (87.0%) | | 0.588 |  |  |
| Patients receiving any rate controlling medication (24 months FU) | | 968/1159 (83.5%) | 1013/1171 (86.5%) | | 1981/2330 (85.0%) | | 0.042 |  |  |
| **Patients receiving diuretics** | | | | | | |  | |  |
| Diuretics (discharge from BL) | | 559/1389 (40.2%) | 561/1393 (40.3%) | | 1120/2782 (40.3%) | | 0.987 |  |  |
| Diuretics (12 months FU) | | 508/1230 (41.3%) | 521/1241 (42.0%) | | 1029/2471 (41.6%) | | 0.788 |  |  |
| Diuretics (24 months FU) | | 478/1159 (41.2%) | 507/1171 (43.3%) | | 985/2330 (42.3%) | | 0.299 |  |  |
| **Patients receiving heart failure and antihypertensive therapy (ACE inhibitor, angiotensin receptor blocker, mineralocorticoid antagonists, and neprilysin/valsartan)** | | | | | | |  | |  |
| Heart failure and antihypertensive therapies (discharge from BL) | | 964/1389 (69.4%) | 988/1393 (70.9%) | | 1952/2782 (70.2%) | | 0.397 |  |  |
| Heart failure and antihypertensive therapies (12 months FU) | | 854/1230 (69.4%) | 878/1241 (70.7%) | | 1732/2471 (70.1%) | | 0.482 |  |  |
| Heart failure and antihypertensive therapies (24 months FU) | | 798/1159 (68.9%) | 837/1171 (71.5%) | | 1635/2330 (70.2%) | | 0.163 |  |  |
| **Patients receiving diabetes therapy (oral antidiabetic medication and insulin)** | | | | | | |  | |  |
| Antidiabetic therapy (discharge from BL) | | 256/1389 (18.4%) | 254/1393 (18.2%) | | 510/2782 (18.3%) | | 0.873 |  |  |
| Antidiabetic therapy (12 months FU) | | 238/1230 (19.3%) | 237/1241 (19.1%) | | 475/2471 (19.2%) | | 0.870 |  |  |
| Antidiabetic therapy (24 months FU) | | 228/1159 (19.7%) | 227/1171 (19.4%) | | 455/2330 (19.5%) | | 0.924 |  |  |
| **Patients receiving Statins** | | | | | | |  | |  |
| Statins (discharge from BL) | | 628/1389 (45.2%) | 568/1393 (40.8%) | | 1196/2782 (43.0%) | | 0.016 |  |  |
| Statins (12 months FU) | | 587/1230 (47.7%) | 526/1241 (42.4%) | | 1113/2471 (45.0%) | | 0.006 |  |  |
| Statins (24 months FU) | | 576/1159 (49.7%) | 529/1171 (45.2%) | | 1105/2330 (47.4%) | | 0.020 |  |  |

**Table 2:** In-person study visits at one, two and three years, triggered and unscheduled visits

|  | **Early treatment** | **Usual care** | **p-value** |
| --- | --- | --- | --- |
| **FU 12 months** | 1230 | 1241 | 0.495* |
| **FU 24 months** | 1159 | 1171 | 0.545* |
| **FU 36 months** | 117 | 119 | 0.849* |
| **Triggered visits total (nr. per patient)** | 249 (0.18) | 93 (0.07) | <0.001** |
| **Unscheduled visits total (nr. per patient)** | 219 (0.16) | 86 (0.06) | <0.001** |
| **Total number of visits total (nr. per patient)** | 2974 (2.13) | 2710 (1.94) | <0.001** |
| *p-value resulting from mixed logistic regression; **p-value resulting from mixed Poisson regression; both models with center as random effect. | | | |

**Table 3:** Apparent violations of class I recommendations for rhythm control therapy use in the EAST – AFNET 4 population. Over 97% of patients received rhythm control therapy in line with recommendations of the ESC guidelines published between 2012 and 2020. ^1, 10, 11^ The most common apparent violation was the use of sodium channel blockers in patients with coronary artery disease (35 patients, 1.3%).

|  | **Randomized group** | |  |  |
| --- | --- | --- | --- | --- |
|  | **Early rhythm control (N=1395)** | **Usual care (N=1394)** | **Total (N=2789)** | **p-value** |
| Severe coronary artery disease in patients receiving flecainide or propafenone at discharge | 32 (2.3%) | 3 (0.2%) | 35 (1.3%) | <0.001* |
| Reduced left ventricular function in patients receiving flecainide or propafenone at discharge | 2 (0.1%) | 1 (0.1%) | 3 (0.1%) | 0.572* |
| Reduced left ventricular function in patients receiving dronedarone at discharge | 3 (0.2%) | 0 (0.0%) | 3 (0.1%) | 0.250** |
| At least one violation of guideline conform use | 37 (2.7%) | 4 (0.3%) | 41 (1.5%) | <0.001* |

*p-value resulting from mixed logistic regression with center as random effect; **p-value resulting from Fisher's exact test.

**Figure 1: Anticoagulation therapy** in patients randomized to early rhythm control (left panel) and usual care (right panel) in the EAST – AFNET 4 population at discharge from randomization, one year, and two years of follow-up. There was no difference in anticoagulation therapy between randomized groups. A combination of both was very rare and therefore the yellow bars are hardly visible.


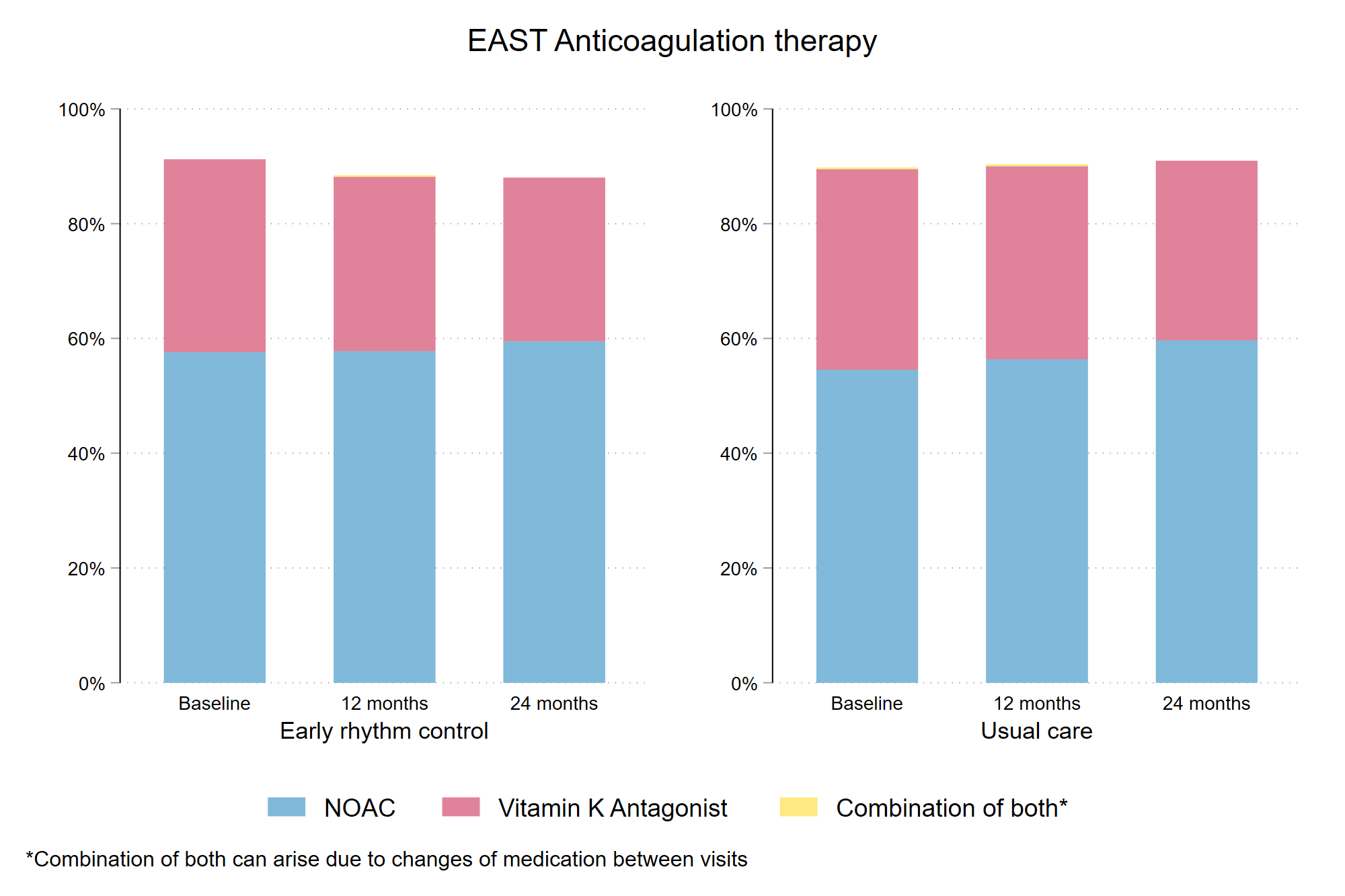


**Figure 2A:** Use of inhibitors of the renin-angiotensin-aldosterone system in patients randomized to early rhythm control (left panel) and usual care (right panel) in the EAST – AFNET 4 population.


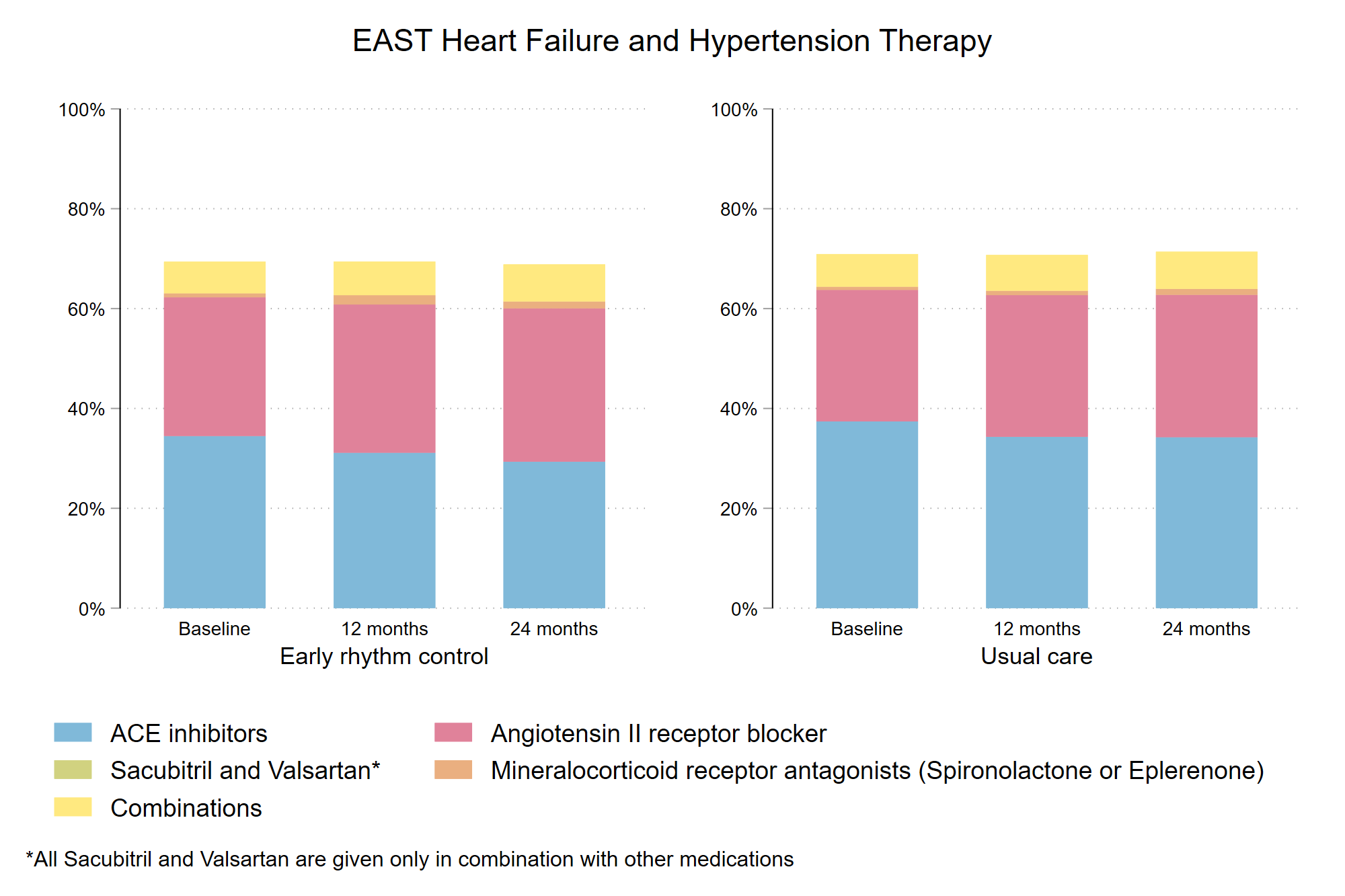


**Figure 2B:** Systolic and diastolic blood pressure during the in-person visits, split by randomized groups. Blood pressure was not different between randomized groups.


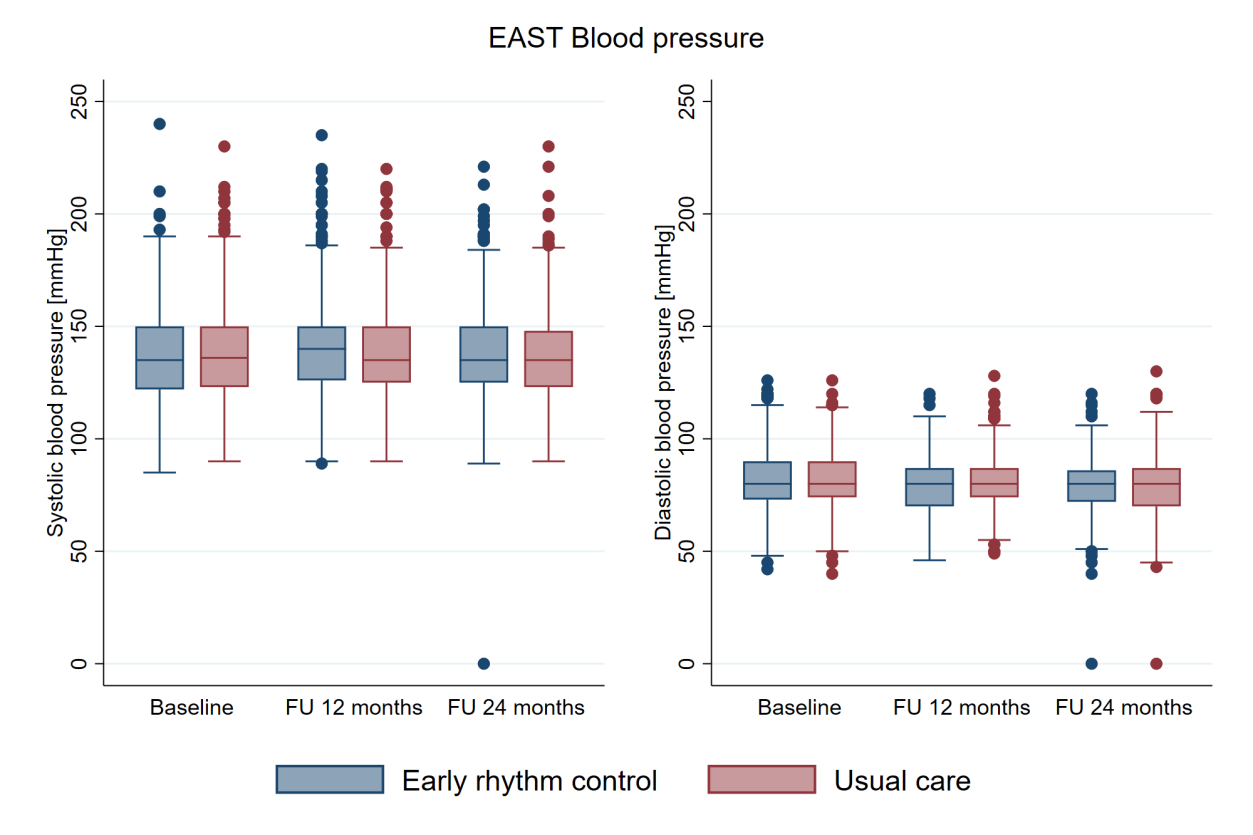


**Figure 3:** Use of any rate controlling therapies in patients randomized to early rhythm control (left panel) and usual care (right panel) in the EAST – AFNET 4 population. This display includes antiarrhythmic drugs with rate controlling properties, namely amiodarone, dronedarone, propafenone, and sotalol. The use of these medications often obviates the need for additional rate-controlling medication, explaining the lower use of beta blockers, calcium channel antagonists, or digoxin shown in Figure 3A.


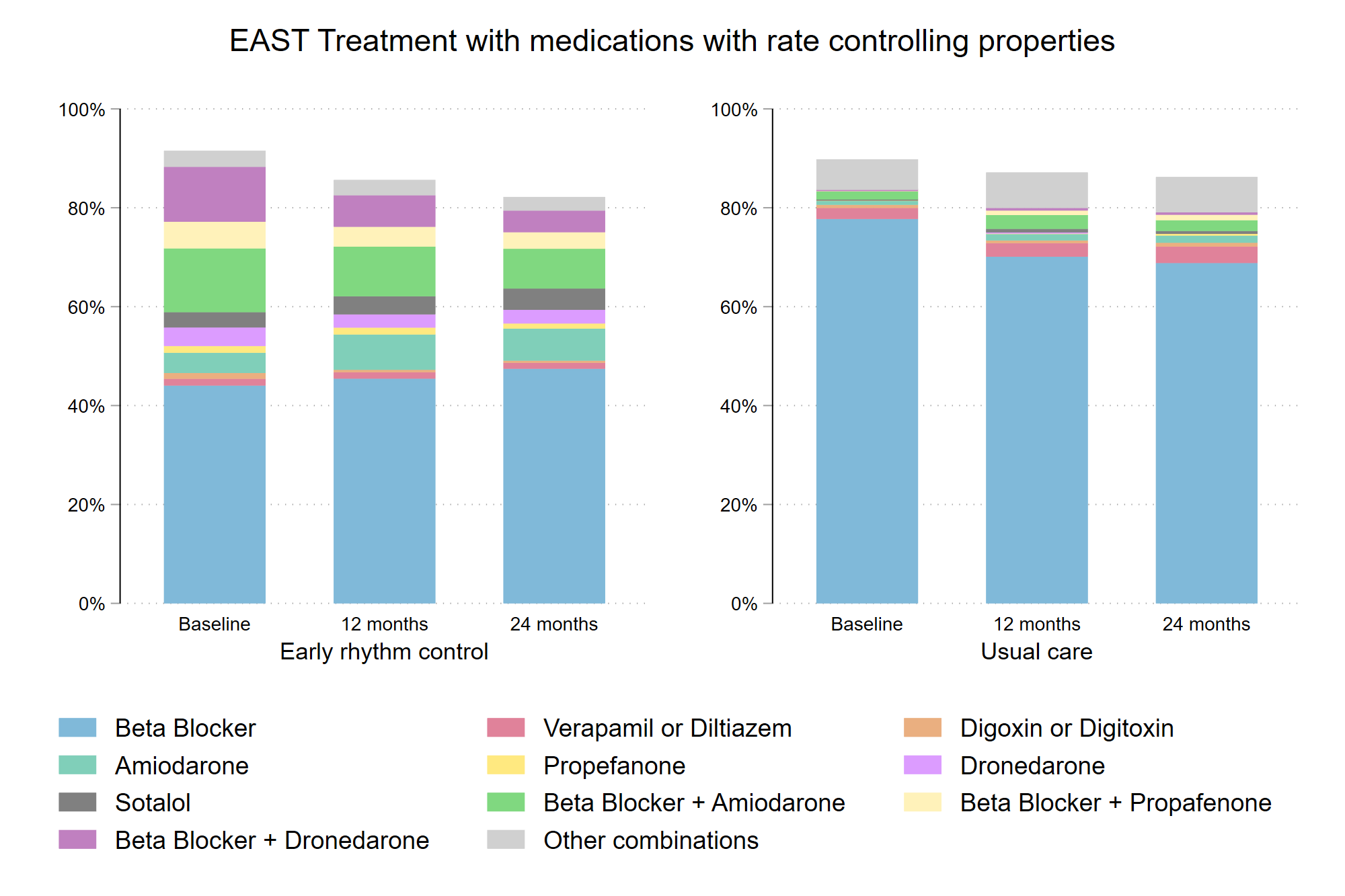


**Figure 4A:** Number of in-person visits split by randomized group. There were 2710 in-person visits in patients randomized to usual care (1.94 visits/patient) and 2974 in-person visits in patients randomized to early rhythm control (2.13 visits/patient) (p<0.001).


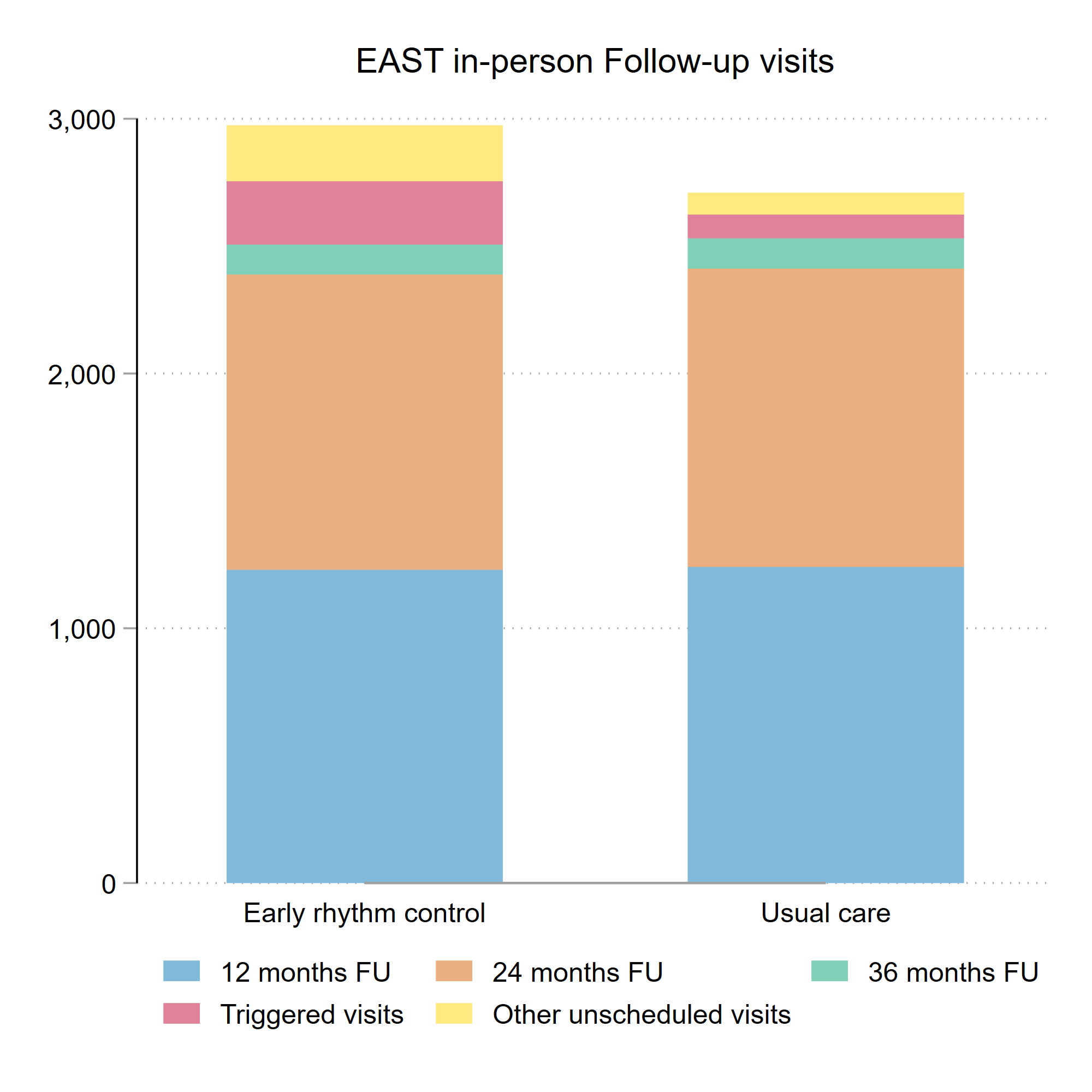


**Figure 4B**: Timing of in-person follow-up visits split by randomized group and by visit type. All numbers are displayed as number of visits per day.


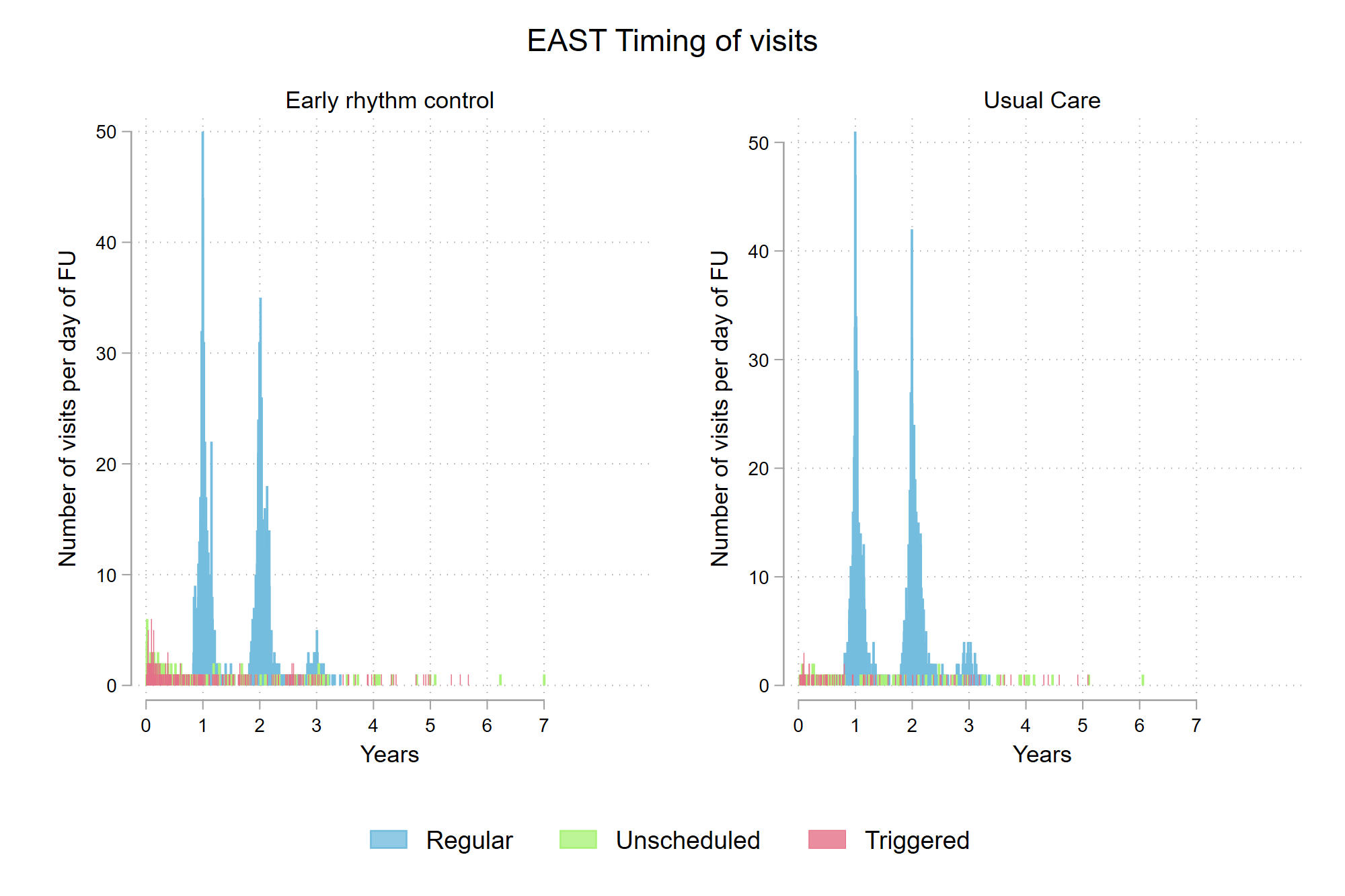


**Figure 5A:** Sankey Plot of rhythm control treatment over time per group. Shown is the proportion of patients receiving antiarrhythmic drugs (AAD) and AF ablation (ablation) at each of the scheduled visits, split by randomized groups, and the proportion of patients changing from one type of therapy to the other.


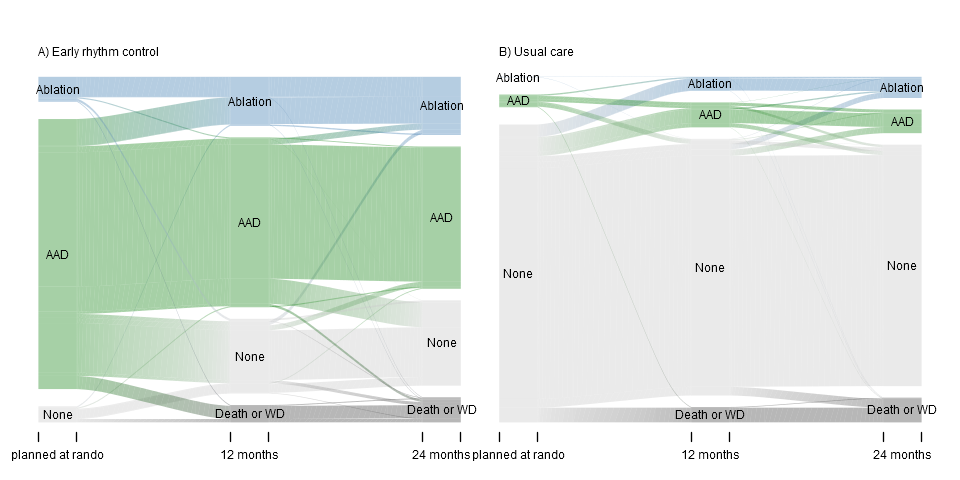


**Figure 5B**: Time to first AF ablation split by randomized group (Aalen-Johansen cumulative incidence curve). AF ablation was more often used in patients randomized to early therapy, with a steady increase in both randomized groups over time. At two years, 270/1395 (19.4%) patients randomized to early therapy had undergone AF ablation, while 97/1394 (7.0%) patients randomized to usual care had undergone ablation.


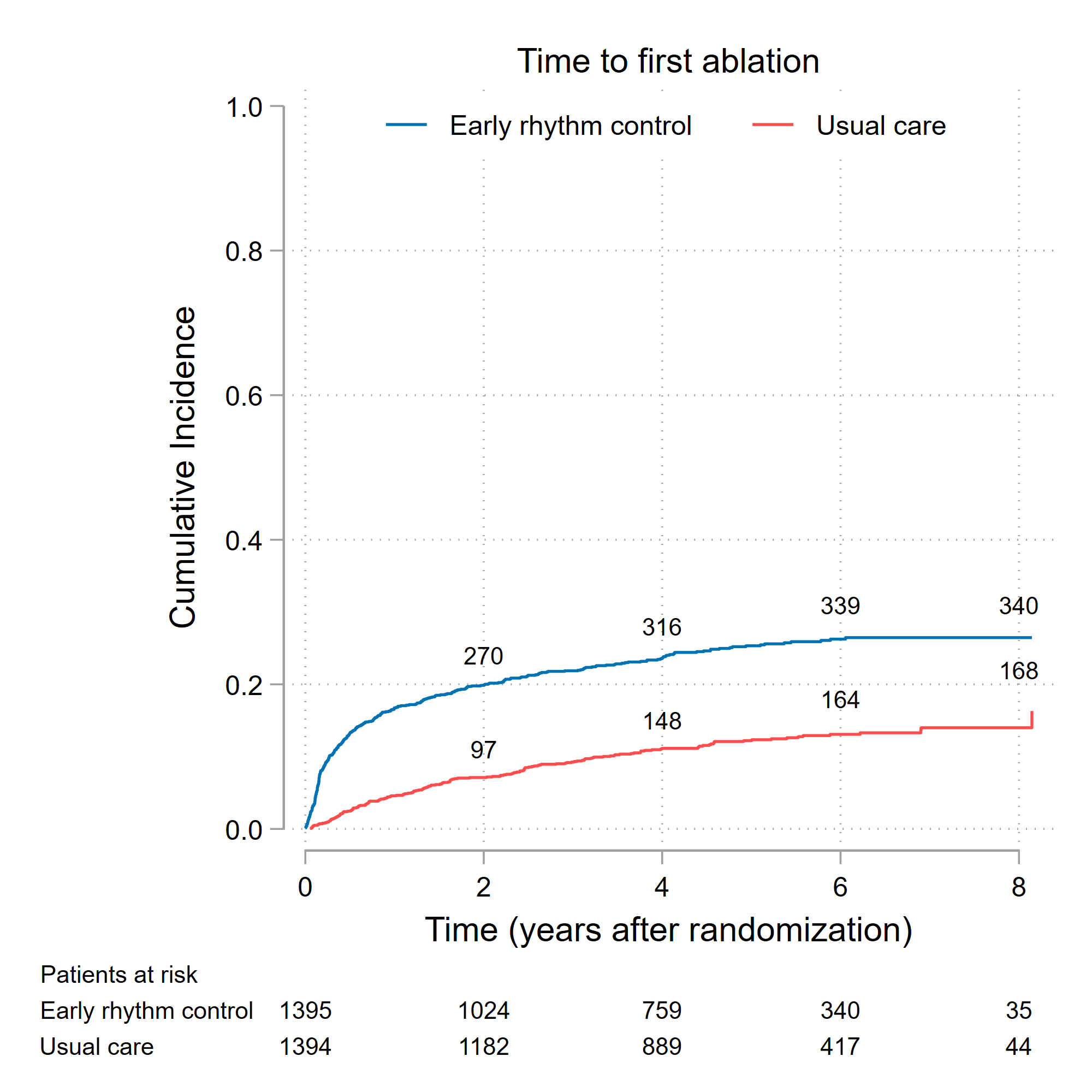


**Figure 6A:** Multivariate analysis of potential factors influencing the decision to manage patients without rhythm control therapy (None, left panel), to perform AF ablation (middle panel), and to initiate antiarrhythmic drug therapy (AAD, right panel) at any time. The decision to manage without rhythm control therapy was almost entirely driven by randomized group. The decision to perform AF ablation was also influenced by younger age, randomization in an ablation site, diabetes, AF pattern, and country. Stable heart failure was defined as either NYHA stage II or LVEF < 50%; TIA=Transient ischemic attack; Severe CAD=Severe coronary artery disease (previous myocardial infarction, CABG or PCI); Left ventricular hypertrophy on echocardiography was defined based on the inclusion criterium (> 15mm wall thickness); AF type first=First episode or paroxysmal, persistent=persistent or long-standing persistent; ET=Early treatment, UC=Usual care.


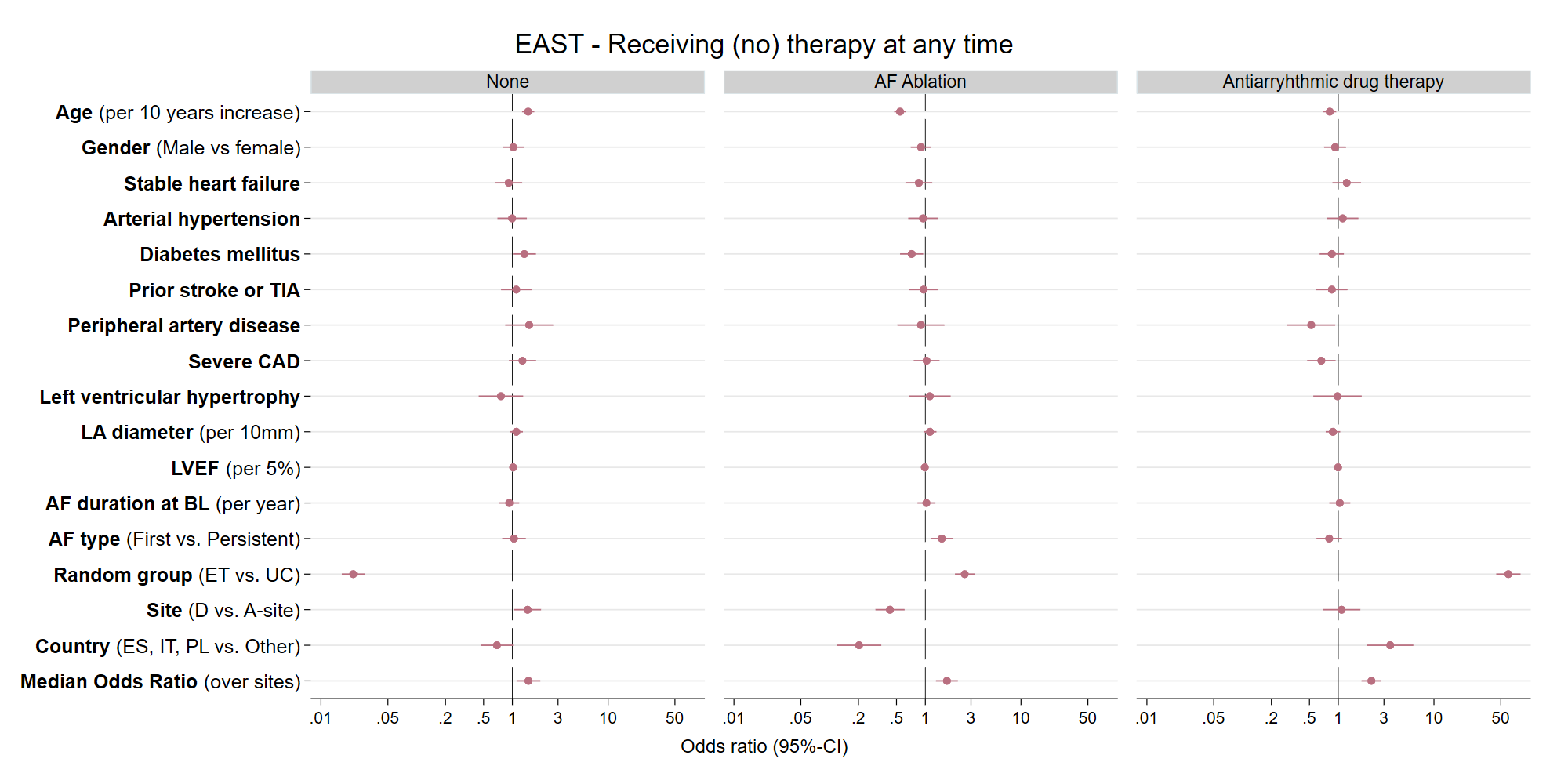


**Figure 6B:** Choice of initial rhythm control therapy displayed by center. Displayed is the proportion of patients receiving each rhythm control therapy option in each center, limited to centers that initiated rhythm control therapy in at least five patients. There are clear center-based preferences in the choice of initial antiarrhythmic drug therapy, with individual sites using AF ablation, flecainide, propafenone, dronedarone, or other antiarrhythmic drugs in most patients initially. Therapy choices were guideline-conform in almost all patients.


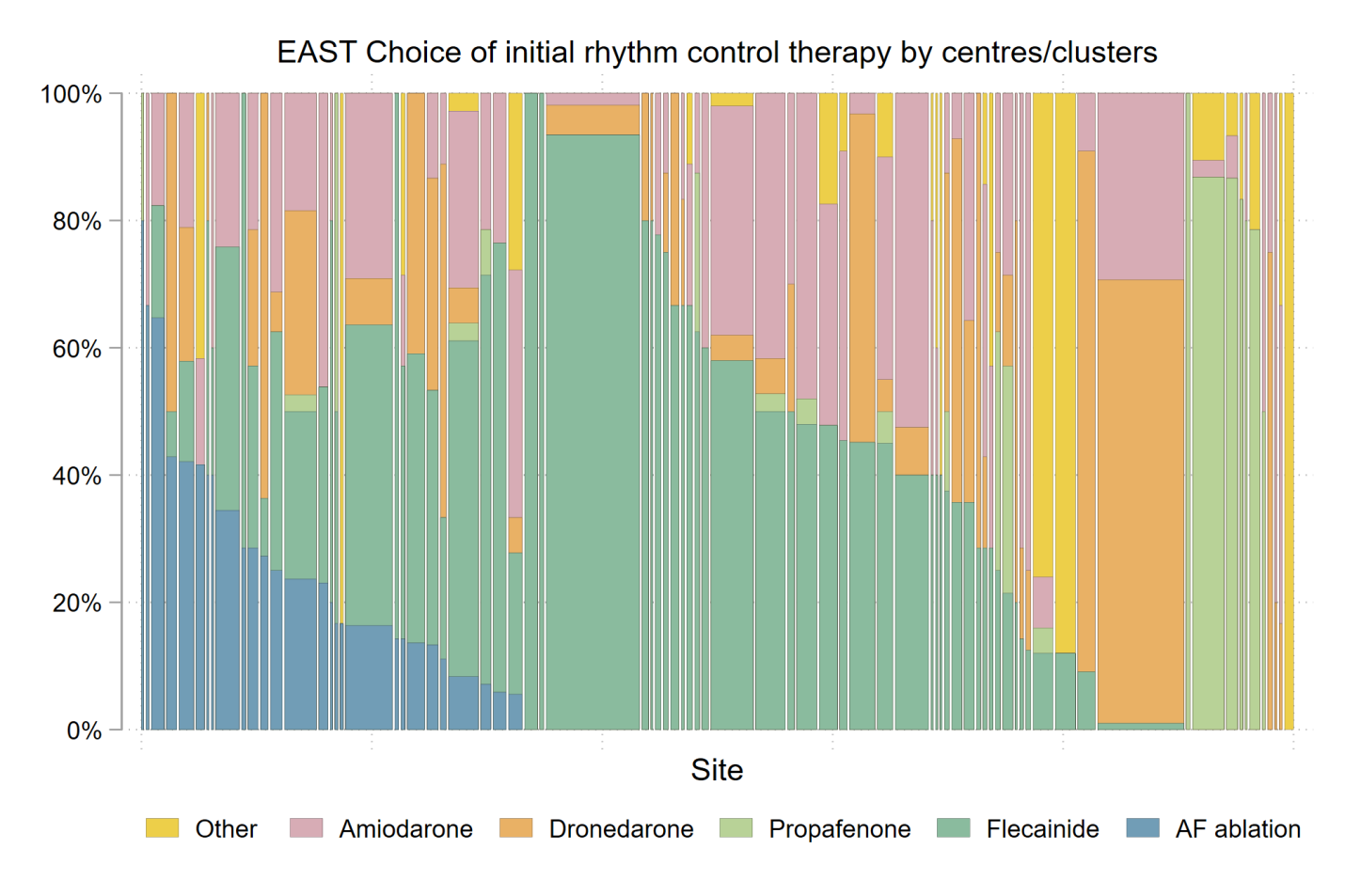


**Supplementary Tables and Figures**

**Supplementary Table 1.** List of study sites

| **Site** | **City** | **Country** | **Type** | **Category** | **Designated A-site** | **Pts. randomized** |
| --- | --- | --- | --- | --- | --- | --- |
| ***UZ Leuven*** | ***Leuven*** | ***be*** | ***University hospital*** | ***A-site*** | ***009*** | ***13*** |
| Practice Dr. L. De Wolf | Tienen | be | Private practice / office based | D-site | 009 | 2 |
| Mariaziekenhuis Noord-Limburg | Overpelt | be | Community hospital | D-site | 009 | 1 |
| Medisch Centrum voor Huisartsen | Leuven | be | Private practice / office based | D-site | 009 | 3 |
| ***OLV Ziekenhuis*** | ***Aalst*** | ***be*** | ***Community hospital*** | ***A-site*** | ***338*** | ***16*** |
| AZ Glorieux, Ronse | Ronse | be | Community hospital | D-site | 338 | 5 |
| OLV Hospital Campus Asse | Asse | be | Community hospital | D-site | 338 | 2 |
| ***Ziekenhuis Oost Limburg*** | ***Genk*** | ***be*** | ***Community hospital*** | ***A-site*** | ***351*** | ***20*** |
| Ziekenhuis Maas en Kempen | Maaseik | be | Community hospital | D-site | 351 | 46 |
| ***AZ Delta Campus Wilgenstraat*** | ***Roeselare*** | ***be*** | ***Community hospital*** | ***A-site*** | ***385*** | ***2*** |
| Sint-Andriesziekenhuis | Tielt | be | Community hospital | D-site | 385 | 15 |
| AZ Delta Campus Brugsesteenweg | Roeselare | be | Community hospital | D-site | 385 | 2 |
| ***Jessa Ziekenhuis*** | ***Hasselt*** | ***be*** | ***Research clinic / academic teaching hospital*** | ***A-site*** | ***419*** | ***10*** |
| Sint-Franciskus Ziekenhuis | Heusden-Zolder | be | Community hospital | D-site | 419 | 6 |
| ***Universitätsspital Zürich*** | ***Zürich*** | ***ch*** | ***University hospital*** | ***A-site*** | ***005*** | ***15*** |
| ***Kantonsspital Luzern*** | ***Luzern 16*** | ***ch*** | ***Community hospital*** | ***A-site*** | ***034*** | ***28*** |
| Kantonsspital Obwalden Sarnen | Sarnen | ch | Community hospital | D-site | 034 | 2 |
| ***Herz-Neuro-Zentrum Bodensee*** | ***Kreuzlingen 2*** | ***ch*** | ***University hospital*** | ***A-site*** | ***045*** | ***0*** |
| Praxis Dr. Bernd Eigenberger | Kreuzlingen | ch | Private practice / office based | D-site | 045 | 1 |
| ***Institute for Clinical and Experimental Medicine (IKEM)*** | ***Prague 4*** | ***cz*** | ***University hospital*** | ***A-site*** | ***017*** | ***13*** |
| ***General University Hospital, First Faculty of Medicine, Charles University*** | ***Prague 2*** | ***cz*** | ***University hospital*** | ***A-site*** | ***180*** | ***10*** |
| General University Hospital | Prague 2 | cz | University hospital | D-site | 180 | 2 |
| ÚVN Military University Hospital Prague | Prague 6 | cz | University hospital | D-site | 180 | 10 |
| ***Universitätsklinikum Münster*** | ***Münster*** | ***de*** | ***University hospital*** | ***A-site*** | ***002*** | ***17*** |
| Kardiologische Praxis Dr. Menz | Menden | de | Private practice / office based | D-site | 002 | 18 |
| ***Universitäres Herzzentrum Hamburg*** | ***Hamburg*** | ***de*** | ***University hospital*** | ***A-site*** | ***003*** | ***30*** |
| Praxis Dr. Jens Beermann | Wedel | de | Private practice / office based | D-site | 003 | 24 |
| Cardiologicum Hamburg | Hamburg | de | Private practice / office based | D-site | 003 | 14 |
| Kardiologische Praxis Hamburg Altona | Hamburg | de | Private practice / office based | D-site | 003 | 3 |
| Kardiologische Praxis HH Altona | Hamburg | de | Private practice / office based | D-site | 003 | 78 |
| Kardiologische-Pneumologische Gemeinschaftspraxis | Hamburg | de | Private practice / office based | D-site | 003 | 1 |
| ***Asklepios Klinik St. Georg*** | ***Hamburg*** | ***de*** | ***Research clinic / academic teaching hospital*** | ***A-site*** | ***004*** | ***69*** |
| Praxis Dr. Hans-Eckart Sarnighausen | Lüneburg | de | Private practice / office based | D-site | 004 | 44 |
| MVZ Prof. Mathey, Prof. Schofer | Hamburg | de | Private practice / office based | D-site | 004 | 2 |
| Cardiomed an der Alster | Hamburg | de | Private practice / office based | D-site | 004 | 9 |
| ***Klinik Augustinum München*** | ***München*** | ***de*** | ***Research clinic / academic teaching hospital*** | ***A-site*** | ***018*** | ***30*** |
| Kardiologische Praxis Dr. Martin Prohaska | Mühldorf am Inn | de | Private practice / office based | D-site | 018 | 19 |
| ***St. Marienhospital Bonn*** | ***Bonn*** | ***de*** | ***Research clinic / academic teaching hospital*** | ***A-site*** | ***028*** | ***72*** |
| St. Johannes-Krankenhaus | Troisdorf | de | Community hospital | D-site | 028 | 24 |
| Marienhospital Brühl | Brühl | de | Community hospital | D-site | 028 | 10 |
| ***Vivantes Klinikum am Urban*** | ***Berlin*** | ***de*** | ***Research clinic / academic teaching hospital*** | ***A-site*** | ***036*** | ***40*** |
| Vivantes Klinikum am Friedrichshain | Berlin | de | Research clinic / academic teaching hospital | D-site | 036 | 66 |
| ***Universitäts-Herzzentrum Freiburg Bad Krozingen GmbH*** | ***Bad Krozingen*** | ***de*** | ***University hospital*** | ***A-site*** | ***037*** | ***0*** |
| Ostalb-Klinikum Aalen | Aalen | de | Research clinic / academic teaching hospital | D-site | 037 | 1 |
| Gemeinschaftspraxis Drs. Thierfelder, Gansser, Rosenthal, Saurbier | Freiburg i. Breisgau | de | Private practice / office based | D-site | 037 | 4 |
| ***Universitätsklinikum Schleswig-Holstein, Campus Lübeck*** | ***Lübeck*** | ***de*** | ***University hospital*** | ***A-site*** | ***038*** | ***14*** |
| DRK Krankenhaus Ratzeburg | Ratzeburg | de | Research clinic / academic teaching hospital | D-site | 038 | 63 |
| DRK Krankenhaus Grevesmühlen | Grevesmühlen | de | Community hospital | D-site | 038 | 6 |
| ***Leipzig Heart Institute GmbH*** | ***Leipzig*** | ***de*** | ***University hospital*** | ***A-site*** | ***049*** | ***108*** |
| Praxis Dr. Jens Taggeselle | Markkleeberg | de | Private practice / office based | D-site | 049 | 216 |
| Praxis Dr. Thomas Peschel | Leipzig | de | Private practice / office based | D-site | 049 | 45 |
| Praxis Dres. Löbe und Weißbrodt | Leipzig | de | Private practice / office based | D-site | 049 | 21 |
| ***St. Vincenz Krankenhaus*** | ***Paderborn*** | ***de*** | ***Research clinic / academic teaching hospital*** | ***A-site*** | ***182*** | ***13*** |
| St. Josefs Krankenhaus | Salzkotten | de | Community hospital | D-site | 182 | 1 |
| Praxis Dr. Jürgen Brunn | Paderborn | de | Private practice / office based | D-site | 182 | 11 |
| ***Herz-Zentrum Bodensee*** | ***Konstanz*** | ***de*** | ***Community hospital*** | ***A-site*** | ***190*** | ***2*** |
| Klinikum Konstanz | Konstanz | de | Research clinic / academic teaching hospital | D-site | 190 | 98 |
| Kardiologische Praxis Dr. Boscher | Biberach an der Riß | de | Private practice / office based | D-site | 190 | 16 |
| ***Med. Klinik und Poliklinik I Grosshadern LMU*** | ***München*** | ***de*** | ***University hospital*** | ***A-site*** | ***263*** | ***40*** |
| Praxis Dr. Norbert Schön | Mühldorf am Inn | de | Private practice / office based | D-site | 263 | 189 |
| Kreisklinik Mindelheim | Mindelheim | de | Community hospital | D-site | 263 | 28 |
| Medizinisches Versorgungszentrum Dachau | Dachau | de | Private practice / office based | D-site | 263 | 6 |
| ***Universitätsklinikum Bonn*** | ***Bonn*** | ***de*** | ***University hospital*** | ***A-site*** | ***307*** | ***79*** |
| Praxis für Kardiologie Bonn | Bonn | de | Private practice / office based | D-site | 307 | 12 |
| Kardiologische Praxis Dr. Andrea Hostert | Bad Neuenahr | de | Private practice / office based | D-site | 307 | 28 |
| ***Städtisches Klinikum Lüneburg*** | ***Lüneburg*** | ***de*** | ***Research clinic / academic teaching hospital*** | ***A-site*** | ***321*** | ***13*** |
| EV Krankenhaus Stift Bethlehem | Ludwigslust | de | Research clinic / academic teaching hospital | D-site | 321 | 3 |
| Städtisches Krankenhaus Kiel | Kiel | de | Research clinic / academic teaching hospital | D-site | 322 | 1 |
| ***Charité Berlin, Campus Virchow-Klinikum*** | ***Berlin*** | ***de*** | ***University hospital*** | ***A-site*** | ***352*** | ***25*** |
| Kardiologische Praxis Rankestraße | Berlin | de | Private practice / office based | D-site | 352 | 1 |
| ***Klinikum Leverkusen*** | ***Leverkusen*** | ***de*** | ***Community hospital*** | ***A-site*** | ***410*** | ***30*** |
| Internistische Gemeinschaftspraxis | Leverkusen | de | Private practice / office based | D-site | 410 | 1 |
| Praxis Dr. Heinemann | Leichlingen | de | Private practice / office based | D-site | 410 | 5 |
| ***Universitätsklinikum Köln*** | ***Köln*** | ***de*** | ***University hospital*** | ***A-site*** | ***411*** | ***1*** |
| ***Klinikum Bielefeld*** | ***Bielefeld*** | ***de*** | ***Community hospital*** | ***A-site*** | ***420*** | ***1*** |
| ***Sana Kliniken Lübeck*** | ***Lübeck*** | ***de*** | ***Community hospital*** | ***A-site*** | ***424*** | ***12*** |
| ***Odense University Hospital*** | ***Odense C*** | ***dk*** | ***University hospital*** | ***A-site*** | ***066*** | ***23*** |
| Sydvestjysk Sygehus Esbjerg | Esbjerg | dk | Community hospital | D-site | 066 | 30 |
| ***Hospital Clinic Barcelona*** | ***Barcelona*** | ***es*** | ***University hospital*** | ***A-site*** | ***014*** | ***41*** |
| Hospital de Sabadell | Sabadell | es | University hospital | D-site | 014 | 8 |
| Hospital Universitari San Joan de Reus | Reus | es | University hospital | D-site | 014 | 16 |
| ***Hospital del Mar*** | ***Barcelona*** | ***es*** | ***University hospital*** | ***A-site*** | ***050*** | ***13*** |
| ***Hospital Universitario Ramón y Cajal*** | ***Madrid*** | ***es*** | ***University hospital*** | ***A-site*** | ***054*** | ***7*** |
| ***Hospital General Universitario De Alicante*** | ***Alicante*** | ***es*** | ***University hospital*** | ***A-site*** | ***057*** | ***3*** |
| Hospital General Universitario Elche | Elche | es | University hospital | D-site | 057 | 1 |
| Hospital Virgen de los Lirios Alcoy | Alcoy | es | Community hospital | D-site | 057 | 16 |
| ***Hospital Clinico San Carlos*** | ***Madrid*** | ***es*** | ***University hospital*** | ***A-site*** | ***063*** | ***18*** |
| ***Fundación Jiménez Diaz*** | ***Madrid*** | ***es*** | ***University hospital*** | ***A-site*** | ***071*** | ***6*** |
| ***Clinique Ambroise Paré*** | ***Neuilly sur Seine*** | ***fr*** | ***Community hospital*** | ***A-site*** | ***021*** | ***1*** |
| ***CHU de Nancy, Hôpitaux de Brabois*** | ***Vandoeuvre les Nancy*** | ***fr*** | ***University hospital*** | ***A-site*** | ***265*** | ***2*** |
| ***University of Leicester, Glenfield General Hospital*** | ***Leicester*** | ***gb*** | ***University hospital*** | ***A-site*** | ***331*** | ***12*** |
| Grantham and District Hospital | Grantham | gb | Community hospital | D-site | 331 | 15 |
| Royal Derby Hospital | Derby | gb | University hospital | D-site | 331 | 1 |
| Kettering General Hospital | Kettering Northamptonshire | gb | Community hospital | D-site | 331 | 39 |
| University Hospital Coventry | Coventry | gb | University hospital | D-site | 331 | 6 |
| ***Queen Elisabeth Hospital Birmingham*** | ***Birmingham*** | ***gb*** | ***University hospital*** | ***A-site*** | ***332*** | ***7*** |
| Royal Stoke University Hospital | Stoke-on-Trent, Staffordshire | gb | University hospital | D-site | 332 | 7 |
| City Hospital Birmingham | Birmingham | gb | University hospital | D-site | 332 | 28 |
| Sandwell General Hospital Birmingham | West Midlands | gb | University hospital | D-site | 332 | 8 |
| ***St. George's Hospital*** | ***London*** | ***gb*** | ***University hospital*** | ***A-site*** | ***360*** | ***5*** |
| ***Wythenshawe University Hospital of South Manchester*** | ***Wythenshawe, Manchester*** | ***gb*** | ***University hospital*** | ***A-site*** | ***374*** | ***0*** |
| The Royal Oldham Hospital | Oldham | gb | Community hospital | D-site | 374 | 12 |
| ***St Bartholomew’s Hospital*** | ***London*** | ***gb*** | ***University hospital*** | ***A-site*** | ***377*** | ***6*** |
| North Middlesex Hospital | London | gb | University hospital | D-site | 377 | 16 |
| Barnet & Chase Farm Hospital | Barnet | gb | Community hospital | D-site | 377 | 11 |
| ***Basildon University Hospital*** | ***Basildon, Essex*** | ***gb*** | ***University hospital*** | ***A-site*** | ***394*** | ***0*** |
| Broomfield Hospital | Broomfield, Chelmsford, Essex | gb | Community hospital | D-site | 394 | 19 |
| Southend University Hospital NHS Foundation Trust | Westcliff-on-Sea, Essex | gb | University hospital | D-site | 394 | 33 |
| The Princess Alexandra Hospital | Harlow, Essex | gb | Community hospital | D-site | 394 | 11 |
| ***Leeds General Infirmary*** | ***Leeds, West Yorkshire*** | ***gb*** | ***University hospital*** | ***A-site*** | ***407*** | ***0*** |
| Airedale General Hospital | Keighley, West Yorkshire | gb | Community hospital | D-site | 407 | 6 |
| Bradford Royal Infirmary | Bradford, West Yorkshire | gb | Community hospital | D-site | 407 | 6 |
| ***Ospedale Dell'Angelo*** | ***Mestre*** | ***it*** | ***Community hospital*** | ***A-site*** | ***205*** | ***5*** |
| Garibaldi-Nesima Hospital | Catania | it | Community hospital | D-site | 205 | 3 |
| ***Azienda Ospedaliera di Padova*** | ***Padova*** | ***it*** | ***Community hospital*** | ***A-site*** | ***206*** | ***14*** |
| ***Ospedale de Circolo e Fondazione Macchi, University of Insubria*** | ***Varese*** | ***it*** | ***University hospital*** | ***A-site*** | ***210*** | ***8*** |
| ***Azienda Ospedaliera S. Maria Nuova*** | ***Reggio Emilia*** | ***it*** | ***Community hospital*** | ***A-site*** | ***213*** | ***22*** |
| Ospedale S. Anna Castelnovo Ne Monti | Castelnovo Ne Monti | it | Community hospital | D-site | 213 | 2 |
| ***Ospedale F. Miulli*** | ***Acquaviva delle Fonti (Bari)*** | ***it*** | ***Community hospital*** | ***A-site*** | ***215*** | ***2*** |
| G. Tatarella | Cerignola (Foggia) | it | Community hospital | D-site | 215 | 2 |
| Ospedale San Paolo | Bari | it | Community hospital | D-site | 215 | 51 |
| ***Policlinico Casilino*** | ***Roma*** | ***it*** | ***Community hospital*** | ***A-site*** | ***266*** | ***0*** |
| University of Rome, La Sapienza | Rome | it | University hospital | D-site | 266 | 14 |
| ***Ospedale Santa Maria Del Prato*** | ***Feltre*** | ***it*** | ***Community hospital*** | ***A-site*** | ***399*** | ***2*** |
| ***Universitair Medisch Center Groningen*** | ***Groningen*** | ***nl*** | ***University hospital*** | ***A-site*** | ***006*** | ***16*** |
| Refaja Ziekenhuis Stadskanaal | Stadskanaal | nl | Community hospital | D-site | 006 | 1 |
| ***University Hospital Maastricht*** | ***Maastricht*** | ***nl*** | ***University hospital*** | ***A-site*** | ***007*** | ***15*** |
| Zuyderland Medisch Centrum | Heerlen | nl | Community hospital | D-site | 007 | 1 |
| ***Leiden University Medical Center (LUMC)*** | ***Leiden*** | ***nl*** | ***University hospital*** | ***A-site*** | ***008*** | ***3*** |
| Bronovo Hospital | Den Haag | nl | Community hospital | D-site | 008 | 4 |
| Spaarne Gasthuis | Haarlem | nl | Community hospital | D-site | 008 | 17 |
| Alrijne Ziekenhuis | Leiderdorp | nl | Community hospital | D-site | 008 | 6 |
| ***Isala Hospital and Diagram Research Zwolle*** | ***Zwolle*** | ***nl*** | ***Community hospital*** | ***A-site*** | ***068*** | ***54*** |
| Gelre Ziekenhuis Zutphen | Zutphen | nl | Community hospital | D-site | 068 | 51 |
| Ziekenhuis St. Jansdal | Harderwijk | nl | Community hospital | D-site | 068 | 11 |
| ***Erasmus MC Rotterdam*** | ***Rotterdam*** | ***nl*** | ***University hospital*** | ***A-site*** | ***082*** | ***0*** |
| Vlietland ziekenhuis Schiedam | Schiedam | nl | Community hospital | D-site | 082 | 1 |
| ***National Institute of Cardiology*** | ***Warsaw*** | ***pl*** | ***University hospital*** | ***A-site*** | ***025*** | ***29*** |
| National Institute of Cardiology, Early Diagnosis Dept. | Warsaw | pl | Community hospital | D-site | 025 | 14 |
| WSPRiTS Meditrans Department of Cardiology | Warsaw | pl | Community hospital | D-site | 025 | 81 |
| National Institute of Cardiology, Department of valvular heart diseases | Warsaw | pl | University hospital | D-site | 025 | 11 |
| Central Clinical Hospital Ministry of Interior Affairs | Warsaw | pl | Community hospital | D-site | 025 | 28 |

**Supplementary Table 2:** Extended list of cardiovascular treatments in the EAST – AFNET 4 trial at discharge from baseline visit, at 12 months, and at 24 months follow-up. This table details substance use. The summarised data are already shown in the main Table 1 in the paper.

**Supplementary Table 2A.** Treatment by randomized group at discharge from baseline visit

|  | **Randomized group** | |  |  |
| --- | --- | --- | --- | --- |
|  | **Early rhythm control (N=1395)** | **Usual care (N=1394)** | **Total (N=2789)** | **p-value** |
| **Oral anticoagulation** | | | |  |
| **Patients receiving anticoagulation** | **1267/1389 (91.2%)** | **1250/1393 (89.7%)** | **2517/2782 (90.5%)** | **0.149*** |
| **Non Vitamin-K-antagonist oral anticoagulants (NOACs)** | | | |  |
| Apixaban | 202/1389 (14.5%) | 188/1393 (13.5%) | 390/2782 (14.0%) | 0.347* |
| Dabigatran | 168/1389 (12.1%) | 159/1393 (11.4%) | 327/2782 (11.8%) | 0.609* |
| Edoxaban | 24/1389 (1.7%) | 25/1393 (1.8%) | 49/2782 (1.8%) | 0.885* |
| Rivaroxaban | 406/1389 (29.2%) | 392/1393 (28.1%) | 798/2782 (28.7%) | 0.521* |
| *All NOACs* | *800/1389 (57.6%)* | *763/1393 (54.8%)* | *1563/2782 (56.2%)* | 0.103* |
| **Vitamin K antagonists** | | | |  |
| Warfarin | 114/1389 (8.2%) | 122/1393 (8.8%) | 236/2782 (8.5%) | 0.643* |
| Phenprocoumon | 225/1389 (16.2%) | 239/1393 (17.2%) | 464/2782 (16.7%) | 0.399* |
| Acenocoumarol | 128/1389 (9.2%) | 129/1393 (9.3%) | 257/2782 (9.2%) | 1.000** |
| *All vitamin K antagonists* | *467/1389 (33.6%)* | *490/1393 (35.2%)* | *957/2782 (34.4%)* | 0.397* |
| **Rate control therapy** | | | |  |
| Beta blockers | 1058/1389 (76.2%) | 1191/1393 (85.5%) | 2249/2782 (80.8%) | <0.001* |
| Verapamil or Diltiazem | 30/1389 (2.2%) | 44/1393 (3.2%) | 74/2782 (2.7%) | 0.094* |
| Digoxin or Digitoxin | 46/1389 (3.3%) | 85/1393 (6.1%) | 131/2782 (4.7%) | <0.001* |
| *All rate control therapies* | *1088/1389 (78.3%)* | *1235/1393 (88.7%)* | *2323/2782 (83.5%)* | *<0.001** |
| **Antiarrhythmic drugs** |  |  |  |  |
| Amiodarone | 240/1389 (17.3%) | 38/1393 (2.7%) | 278/2782 (10.0%) | <0.001* |
| Propafenone | 94/1389 (6.8%) | 4/1393 (0.3%) | 98/2782 (3.5%) | <0.001* |
| Dronedarone | 210/1389 (15.1%) | 1/1393 (0.1%) | 211/2782 (7.6%) | <0.001* |
| Sotalol | 52/1389 (3.7%) | 1/1393 (0.1%) | 53/2782 (1.9%) | <0.001* |
| Flecainide | 456/1389 (32.8%) | 26/1393 (1.9%) | 482/2782 (17.3%) | <0.001* |
| All antiarrhythmic drugs (discharge from BL) | 1051/1389 (75.7%) | 70/1393 (5.0%) | 1121/2782 (40.3%) | <0.001* |
| **Diuretics** | | | |  |
| Diuretics (loop diuretics) | 234/1389 (16.8%) | 233/1393 (16.7%) | 467/2782 (16.8%) | 0.936* |
| Other diuretcs (incl. thiacide diuretics) | 356/1389 (25.6%) | 364/1393 (26.1%) | 720/2782 (25.9%) | 0.788* |
| *All diuretics* | *559/1389 (40.2%)* | *561/1393 (40.3%)* | *1120/2782 (40.3%)* | *0.987** |
| **Heart failure and antihypertensive therapy** | | | |  |
| ACE inhibitors | 536/1389 (38.6%) | 586/1393 (42.1%) | 1122/2782 (40.3%) | 0.061* |
| Angiotensin II receptor blocker | 426/1389 (30.7%) | 401/1393 (28.8%) | 827/2782 (29.7%) | 0.269* |
| Sacubitril and Valsartan | 0/1389 (0.0%) | 0/1393 (0.0%) | 0/2782 (0.0%) | - |
| Spironolactone or Eplerenone) | 90/1389 (6.5%) | 92/1393 (6.6%) | 182/2782 (6.5%) | 0.891* |
| *All heart failure and antihyp. therapies* | *964/1389 (69.4%)* | *988/1393 (70.9%)* | *1952/2782 (70.2%)* | *0.397** |
| **Diabetes therapy** | | | |  |
| Insulin | 62/1389 (4.5%) | 59/1393 (4.2%) | 121/2782 (4.3%) | 0.782* |
| Metformin | 206/1389 (14.8%) | 197/1393 (14.1%) | 403/2782 (14.5%) | 0.585* |
| Other Antidiabetics | 88/1389 (6.3%) | 113/1393 (8.1%) | 201/2782 (7.2%) | 0.071* |
| All Antidiabetics | 256/1389 (18.4%) | 254/1393 (18.2%) | 510/2782 (18.3%) | 0.873* |
| **Statins** | | | |  |
| Atorvastatin | 169/1389 (12.2%) | 150/1393 (10.8%) | 319/2782 (11.5%) | 0.245* |
| Fluvastatin | 11/1389 (0.8%) | 9/1393 (0.6%) | 20/2782 (0.7%) | 0.654* |
| Lovastatin | 0/1389 (0.0%) | 3/1393 (0.2%) | 3/2782 (0.1%) | 0.250** |
| Pravastatin | 17/1389 (1.2%) | 24/1393 (1.7%) | 41/2782 (1.5%) | 0.273* |
| Rosuvastatin | 24/1389 (1.7%) | 47/1393 (3.4%) | 71/2782 (2.6%) | 0.004* |
| Simvastatin | 407/1389 (29.3%) | 335/1393 (24.0%) | 742/2782 (26.7%) | 0.001* |
| *All Statins* | *628/1389 (45.2%)* | *568/1393 (40.8%)* | *1196/2782 (43.0%)* | *0.016** |

*p-value resulting from mixed logistic regression with center as random effect; **p-value resulting from Fisher's exact test if mixed logistic model was not applicable.

**Supplementary Table 2B**: Therapy at one year

|  | **Randomized group** | |  |  |
| --- | --- | --- | --- | --- |
|  | **Early rhythm control (N=1395)** | **Usual care (N=1394)** | **Total (N=2789)** | **p-value** |
| **Anticoagulation** | | | |  |
| Apixaban | 208/1230 (16.9%) | 183/1241 (14.7%) | 391/2471 (15.8%) | 0.091* |
| Dabigatran | 127/1230 (10.3%) | 127/1241 (10.2%) | 254/2471 (10.3%) | 0.985* |
| Edoxaban | 25/1230 (2.0%) | 34/1241 (2.7%) | 59/2471 (2.4%) | 0.215* |
| Rivaroxaban | 355/1230 (28.9%) | 361/1241 (29.1%) | 716/2471 (29.0%) | 0.722* |
| NOACs | 713/1230 (58.0%) | 704/1241 (56.7%) | 1417/2471 (57.3%) | 0.657* |
| **Vitamin K antagonists** | | | |  |
| Warfarin | 87/1230 (7.1%) | 102/1241 (8.2%) | 189/2471 (7.6%) | 0.373* |
| Phenprocoumon | 183/1230 (14.9%) | 217/1241 (17.5%) | 400/2471 (16.2%) | 0.077* |
| Acenocoumarol | 106/1230 (8.6%) | 102/1241 (8.2%) | 208/2471 (8.4%) | 0.772** |
| All vitamin K antagonists | 376/1230 (30.6%) | 421/1241 (33.9%) | 797/2471 (32.3%) | 0.100* |
| All anticoagulations | 1087/1230 (88.4%) | 1121/1241 (90.3%) | 2208/2471 (89.4%) | 0.111* |
| **Rate control therapy** | | | |  |
| Beta blockers | 851/1230 (69.2%) | 1007/1241 (81.1%) | 1858/2471 (75.2%) | <0.001* |
| Verapamil or Diltiazem | 25/1230 (2.0%) | 54/1241 (4.4%) | 79/2471 (3.2%) | 0.001* |
| Digoxin or Digitoxin | 30/1230 (2.4%) | 83/1241 (6.7%) | 113/2471 (4.6%) | <0.001* |
| All rate control therapies | 883/1230 (71.8%) | 1055/1241 (85.0%) | 1938/2471 (78.4%) | <0.001* |
| **Antiarrhythmic drugs** |  |  |  |  |
| Amiodarone | 226/1230 (18.4%) | 54/1241 (4.4%) | 280/2471 (11.3%) | <0.001* |
| Propafenone | 71/1230 (5.8%) | 15/1241 (1.2%) | 86/2471 (3.5%) | <0.001* |
| Dronedarone | 120/1230 (9.8%) | 8/1241 (0.6%) | 128/2471 (5.2%) | <0.001* |
| Sotalol | 55/1230 (4.5%) | 12/1241 (1.0%) | 67/2471 (2.7%) | <0.001* |
| Flecainide | 361/1230 (29.3%) | 40/1241 (3.2%) | 401/2471 (16.2%) | <0.001* |
| All antiarrhythmic drugs (12 months FU) | 822/1230 (66.8%) | 129/1241 (10.4%) | 951/2471 (38.5%) | <0.001* |
| **Diuretics** | | | |  |
| Diuretics (loop diuretics) | 240/1230 (19.5%) | 240/1241 (19.3%) | 480/2471 (19.4%) | 0.866* |
| Other diuretics (incl. thiacide diuretics) | 301/1230 (24.5%) | 327/1241 (26.3%) | 628/2471 (25.4%) | 0.303* |
| All diuretics | 508/1230 (41.3%) | 521/1241 (42.0%) | 1029/2471 (41.6%) | 0.788* |
| **Heart failure and antihypertensive therapy** | | | |  |
| ACE inhibitors | 429/1230 (34.9%) | 484/1241 (39.0%) | 913/2471 (36.9%) | 0.033* |
| Angiotensin II receptor blocker | 410/1230 (33.3%) | 389/1241 (31.3%) | 799/2471 (32.3%) | 0.289* |
| Sacubitril and Valsartan | 1/1230 (0.1%) | 0/1241 (0.0%) | 1/2471 (0.0%) | 0.498** |
| Mineralocorticoid receptor antagonists (Spironolactone or Eplerenone) | 98/1230 (8.0%) | 95/1241 (7.7%) | 193/2471 (7.8%) | 0.784* |
| All heart failure and antihyp. therapies | 854/1230 (69.4%) | 878/1241 (70.7%) | 1732/2471 (70.1%) | 0.482* |
| **Diabetes therapy** | | | |  |
| Insulin | 56/1230 (4.6%) | 57/1241 (4.6%) | 113/2471 (4.6%) | 0.918* |
| Metformin | 186/1230 (15.1%) | 191/1241 (15.4%) | 377/2471 (15.3%) | 0.849* |
| Other Antidiabetics | 76/1230 (6.2%) | 106/1241 (8.5%) | 182/2471 (7.4%) | 0.025* |
| All Antidiabetics | 238/1230 (19.3%) | 237/1241 (19.1%) | 475/2471 (19.2%) | 0.870* |
| **Statins** | | | |  |
| Atorvastatin | 159/1230 (12.9%) | 152/1241 (12.2%) | 311/2471 (12.6%) | 0.629* |
| Fluvastatin | 11/1230 (0.9%) | 6/1241 (0.5%) | 17/2471 (0.7%) | 0.222* |
| Lovastatin | 0/1230 (0.0%) | 2/1241 (0.2%) | 2/2471 (0.1%) | 0.500** |
| Pravastatin | 20/1230 (1.6%) | 17/1241 (1.4%) | 37/2471 (1.5%) | 0.587* |
| Rosuvastatin | 28/1230 (2.3%) | 45/1241 (3.6%) | 73/2471 (3.0%) | 0.027* |
| Simvastatin | 369/1230 (30.0%) | 304/1241 (24.5%) | 673/2471 (27.2%) | 0.002* |
| All Statins | 587/1230 (47.7%) | 526/1241 (42.4%) | 1113/2471 (45.0%) | 0.006* |

**Supplementary Table 2C**: Therapy at two years

|  | **Randomized group** | |  |  |
| --- | --- | --- | --- | --- |
|  | **Early rhythm control (N=1395)** | **Usual care (N=1394)** | **Total (N=2789)** | **p-value** |
| **Anticoagulation** | | | |  |
| Apixaban | 208/1159 (17.9%) | 199/1171 (17.0%) | 407/2330 (17.5%) | 0.511* |
| Dabigatran | 121/1159 (10.4%) | 119/1171 (10.2%) | 240/2330 (10.3%) | 0.893* |
| Edoxaban | 28/1159 (2.4%) | 36/1171 (3.1%) | 64/2330 (2.7%) | 0.288* |
| Rivaroxaban | 333/1159 (28.7%) | 346/1171 (29.5%) | 679/2330 (29.1%) | 0.576* |
| NOACs | 690/1159 (59.5%) | 699/1171 (59.7%) | 1389/2330 (59.6%) | 0.774* |
| **Vitamin K antagonists** | | | |  |
| Warfarin | 70/1159 (6.0%) | 86/1171 (7.3%) | 156/2330 (6.7%) | 0.327* |
| Phenprocoumon | 167/1159 (14.4%) | 185/1171 (15.8%) | 352/2330 (15.1%) | 0.369* |
| Acenocoumarol | 93/1159 (8.0%) | 95/1171 (8.1%) | 188/2330 (8.1%) | 0.940** |
| All vitamin K antagonists | 330/1159 (28.5%) | 366/1171 (31.3%) | 696/2330 (29.9%) | 0.202* |
| All anticoagulations | 1020/1159 (88.0%) | 1065/1171 (90.9%) | 2085/2330 (89.5%) | 0.021* |
| **Rate control therapy** | | | |  |
| Beta blockers | 777/1159 (67.0%) | 931/1171 (79.5%) | 1708/2330 (73.3%) | <0.001* |
| Verapamil or Diltiazem | 21/1159 (1.8%) | 61/1171 (5.2%) | 82/2330 (3.5%) | <0.001* |
| Digoxin or Digitoxin | 31/1159 (2.7%) | 80/1171 (6.8%) | 111/2330 (4.8%) | <0.001* |
| All rate control therapies | 799/1159 (68.9%) | 986/1171 (84.2%) | 1785/2330 (76.6%) | <0.001* |
| **Antiarrhythmic drugs** |  |  |  |  |
| Amiodarone | 181/1159 (15.6%) | 46/1171 (3.9%) | 227/2330 (9.7%) | <0.001* |
| Propafenone | 55/1159 (4.7%) | 18/1171 (1.5%) | 73/2330 (3.1%) | <0.001* |
| Dronedarone | 89/1159 (7.7%) | 6/1171 (0.5%) | 95/2330 (4.1%) | <0.001* |
| Sotalol | 52/1159 (4.5%) | 8/1171 (0.7%) | 60/2330 (2.6%) | <0.001* |
| Flecainide | 308/1159 (26.6%) | 44/1171 (3.8%) | 352/2330 (15.1%) | <0.001* |
| All antiarrhythmic drugs (24 months FU) | 685/1159 (59.1%) | 120/1171 (10.2%) | 805/2330 (34.5%) | <0.001* |
| **Diuretics** | | | |  |
| Diuretics (loop diuretics) | 228/1159 (19.7%) | 242/1171 (20.7%) | 470/2330 (20.2%) | 0.552* |
| Other diuretcs (incl. thiacide diuretics) | 280/1159 (24.2%) | 306/1171 (26.1%) | 586/2330 (25.2%) | 0.244* |
| All diuretics | 478/1159 (41.2%) | 507/1171 (43.3%) | 985/2330 (42.3%) | 0.299* |
| **Heart failure and antihypertensive therapy** | | | |  |
| ACE inhibitors | 390/1159 (33.6%) | 454/1171 (38.8%) | 844/2330 (36.2%) | 0.011* |
| Angiotensin II receptor blocker | 400/1159 (34.5%) | 373/1171 (31.9%) | 773/2330 (33.2%) | 0.189* |
| Sacubitril and Valsartan | 4/1159 (0.3%) | 2/1171 (0.2%) | 6/2330 (0.3%) | 0.416* |
| Mineralocorticoid receptor antagonists (Spironolactone or Eplerenone) | 92/1159 (7.9%) | 94/1171 (8.0%) | 186/2330 (8.0%) | 0.941* |
| All heart failure and antihyp. therapies | 798/1159 (68.9%) | 837/1171 (71.5%) | 1635/2330 (70.2%) | 0.163* |
| **Diabetes therapy** | | | |  |
| Insulin | 54/1159 (4.7%) | 52/1171 (4.4%) | 106/2330 (4.5%) | 0.917* |
| Metformin | 179/1159 (15.4%) | 174/1171 (14.9%) | 353/2330 (15.2%) | 0.735* |
| Other Antidiabetics | 74/1159 (6.4%) | 105/1171 (9.0%) | 179/2330 (7.7%) | 0.019* |
| All Antidiabetics | 228/1159 (19.7%) | 227/1171 (19.4%) | 455/2330 (19.5%) | 0.924* |
| **Statins** | | | |  |
| Atorvastatin | 177/1159 (15.3%) | 172/1171 (14.7%) | 349/2330 (15.0%) | 0.710* |
| Fluvastatin | 10/1159 (0.9%) | 6/1171 (0.5%) | 16/2330 (0.7%) | 0.335* |
| Lovastatin | 1/1159 (0.1%) | 2/1171 (0.2%) | 3/2330 (0.1%) | 0.577* |
| Pravastatin | 24/1159 (2.1%) | 19/1171 (1.6%) | 43/2330 (1.8%) | 0.422* |
| Rosuvastatin | 31/1159 (2.7%) | 44/1171 (3.8%) | 75/2330 (3.2%) | 0.091* |
| Simvastatin | 334/1159 (28.8%) | 286/1171 (24.4%) | 620/2330 (26.6%) | 0.011* |
| All Statins | 576/1159 (49.7%) | 529/1171 (45.2%) | 1105/2330 (47.4%) | 0.020* |

*p-value resulting from mixed logistic regression with center as random effect; **p-value resulting from Fisher's exact test if mixed logistic model was not applicable.

**Supplementary Table 3:** Number of patients with more than one ablation

|  | **Early treatment  (N=1395)** | **Usual care  (N=1394)** | **Total (N=2789)** |
| --- | --- | --- | --- |
| **0 abl.** | 1055 (75.63%) | 1224 (87.80%) | 2279 (81.71%) |
| **1 abl.** | 242 (17.35%) | 136 (9.76%) | 378 (13.55%) |
| **2 abl.** | 74 (5.30%) | 26 (1.87%) | 100 (3.59%) |
| **3 abl.** | 22 (1.58%) | 6 (0.43%) | 28 (1.00%) |
| **4 abl.** | 1 (0.07%) | 2 (0.14%) | 3 (0.11%) |
| **6 abl.** | 1 (0.07%) | 0 (0.00%) | 1 (0.04%) |

**Supplementary Table 4:** Mixed logistic regressions for therapy (at any time). Of 2789 patients 508 patients received an ablation at any time while on observation, 1373 received AAD medication and 1208 did not receive ablation or medication at any time. The models are based on 2393 patients with complete data (85.8%). For each outcome (ablation, AAD, no therapy) a separate mixed logistic regression model was calculated, including a random effect for center. We did this to account for patients receiving ablation and AAD at different periods. BIC is a measure of model fit and the smaller the BIC gets, the better the model fits. BIC f- shows the loss of model fit when excluding the specific variable from the full model. It measures the independent contribution of a variable. Thus, high values of BIC f- indicate a high importance of a factor for the specific therapeutic decision. Whether the factor increases or decreases the likelihood of an intervention cannot be read off from BIC f-, but can be derived of the Odds Ratio (OR) being >1 or <1, respectively.

|  | **Ablation at any time** | | | | | **AAD at any time** | | | | | **No ablation or AAD** | | | | |
| --- | --- | --- | --- | --- | --- | --- | --- | --- | --- | --- | --- | --- | --- | --- | --- |
|  | **OR** | **95%-CI** | | **p** | **BIC f-** | **OR** | **95%-CI** | | **p** | **BIC f-** | **OR** | **95%-CI** | | **p** | **BIC f-** |
| **Age (per 10 years increase)** | 0.55 | 0.47 | 0.63 | <0.001 | 64 | 0.82 | 0.70 | 0.95 | 0.009 | -1 | 1.46 | 1.26 | 1.70 | <0.001 | 18 |
| **Gender (Male vs female)** | 0.90 | 0.70 | 1.15 | 0.409 | -7 | 0.93 | 0.71 | 1.20 | 0.562 | -7 | 1.02 | 0.80 | 1.32 | 0.858 | -8 |
| **Stable heart failure (NYHA stage II or LVEF < 50%)** | 0.86 | 0.62 | 1.18 | 0.346 | -7 | 1.22 | 0.87 | 1.73 | 0.248 | -6 | 0.92 | 0.66 | 1.27 | 0.599 | -8 |
| **Arterial hypertension** | 0.95 | 0.66 | 1.36 | 0.774 | -8 | 1.11 | 0.76 | 1.62 | 0.578 | -7 | 0.99 | 0.70 | 1.42 | 0.978 | -8 |
| **Diabetes mellitus** | 0.72 | 0.55 | 0.95 | 0.020 | -2 | 0.85 | 0.64 | 1.14 | 0.292 | -7 | 1.34 | 1.01 | 1.76 | 0.040 | -4 |
| **Prior stroke or transient ischemic attack** | 0.96 | 0.68 | 1.35 | 0.814 | -8 | 0.86 | 0.59 | 1.25 | 0.424 | -7 | 1.10 | 0.76 | 1.59 | 0.615 | -8 |
| **Peripheral artery disease** | 0.90 | 0.51 | 1.59 | 0.716 | -8 | 0.52 | 0.29 | 0.93 | 0.027 | -3 | 1.50 | 0.84 | 2.67 | 0.169 | -6 |
| **Severe coronary artery disease (previous myocardial infarction, CABG or PCI)** | 1.03 | 0.76 | 1.41 | 0.845 | -8 | 0.67 | 0.47 | 0.94 | 0.020 | -2 | 1.28 | 0.92 | 1.77 | 0.142 | -6 |
| **Left ventricular hypertrophy on echocardiography (> 15mm wall thickness)** | 1.12 | 0.68 | 1.84 | 0.668 | -8 | 0.98 | 0.55 | 1.76 | 0.949 | -8 | 0.76 | 0.45 | 1.30 | 0.315 | -7 |
| **LA diameter (per 10mm)** | 1.12 | 0.96 | 1.31 | 0.150 | -6 | 0.88 | 0.74 | 1.05 | 0.150 | -6 | 1.10 | 0.94 | 1.29 | 0.241 | -6 |
| **LVEF (per 5%)** | 0.99 | 0.92 | 1.06 | 0.746 | -8 | 0.99 | 0.92 | 1.07 | 0.899 | -8 | 1.02 | 0.95 | 1.09 | 0.577 | -7 |
| **AF duration at BL (per year)** | 1.03 | 0.83 | 1.27 | 0.819 | -8 | 1.04 | 0.80 | 1.33 | 0.786 | -8 | 0.93 | 0.73 | 1.18 | 0.540 | -7 |
| **AF pattern (Persistent or long-standing persistent vs. First episode or paroxysmal)** | 1.49 | 1.14 | 1.95 | 0.004 | 0 | 0.8 | 0.59 | 1.09 | 0.163 | -6 | 1.04 | 0.78 | 1.38 | 0.784 | -8 |
| **Randomized group (Early treatment vs. Usual care)** | 2.58 | 2.04 | 3.27 | <0.001 | 59 | 59.9 | 44.75 | 80.26 | <0.001 | 1413 | 0.02 | 0.02 | 0.03 | <0.001 | 1314 |
| **D vs. A-site** | 0.43 | 0.30 | 0.61 | <0.001 | 12 | 1.08 | 0.69 | 1.70 | 0.729 | -8 | 1.44 | 1.04 | 2.00 | 0.026 | -3 |
| **Country (ES, IT, PL vs. Other)** | 0.20 | 0.12 | 0.35 | <0.001 | 28 | 3.49 | 2.00 | 6.09 | <0.001 | 10 | 0.69 | 0.47 | 1.02 | 0.061 | -4 |
| **Median Odds Ratio (over sites)** | 1.68 | 1.29 | 2.19 | <0.001 | . | 2.22 | 1.75 | 2.82 | <0.001 | . | 1.47 | 1.11 | 1.96 | 0.008 | . |
